# AXL signaling in cancer: from molecular insights to targeted therapies

**DOI:** 10.1038/s41392-024-02121-7

**Published:** 2025-02-10

**Authors:** Monika Yadav, Akansha Sharma, Ketki Patne, Saba Tabasum, Jyoti Suryavanshi, Laxminarayan Rawat, Marc Machaalani, Marc Eid, Rana P. Singh, Toni K. Choueiri, Soumitro Pal, Akash Sabarwal

**Affiliations:** 1 Cancer Biology Laboratory, School of Life Sciences, Jawaharlal Nehru University, New Delhi, Delhi India; 2 Laboratory of Nanotechnology and Chemical Biology, Regional Center for Biotechnology, Faridabad, Haryana India; 3 Department of Microbiology and Immunology, Thomas Jefferson University, Philadelphia, PA USA; 4 Chromatin Remodeling Laboratory, School of Life Sciences, Jawaharlal Nehru University, New Delhi, India; 5 Dana-Farber Cancer Institute, Boston, MA USA; 6 Harvard Medical School, Boston, MA USA; 7 Department of Cell Biology and Anatomy, New York Medical College, Valhalla, NY USA; 8 Division of Nephrology, Boston Children’s Hospital, Boston, MA USA

**Keywords:** Cancer therapy, Oncogenes

## Abstract

AXL, a member of the TAM receptor family, has emerged as a potential target for advanced-stage human malignancies. It is frequently overexpressed in different cancers and plays a significant role in various tumor-promoting pathways, including cancer cell proliferation, invasion, metastasis, epithelial–mesenchymal transition (EMT), angiogenesis, stemness, DNA damage response, acquired therapeutic resistance, immunosuppression, and inflammatory responses. Beyond oncology, AXL also facilitates viral infections, including SARS-CoV-2 and Zika highlighting its importance in both cancer and virology. In preclinical models, small-molecule kinase inhibitors targeting AXL have shown promising anti-tumorigenic potential. This review primarily focuses on the induction, regulation and biological functions of AXL in mediating these tumor-promoting pathways. We discuss a range of therapeutic strategies, including recently developed small-molecule tyrosine kinase inhibitors (TKIs), monoclonal antibodies, and antibody–drug conjugates (ADCs), anti-AXL-CAR, and combination therapies. These interventions are being examined in both preclinical and clinical studies, offering the potential for improved drug sensitivity and therapeutic efficacy. We further discuss the mechanisms of acquired therapeutic resistance, particularly the crosstalk between AXL and other critical receptor tyrosine kinases (RTKs) such as c-MET, EGFR, HER2/HER3, VEGFR, PDGFR, and FLT3. Finally, we highlight key research areas that require further exploration to enhance AXL-mediated therapeutic approaches for improved clinical outcomes.

## Introduction

Cancer progression is significantly influenced by aberrant signaling through receptor tyrosine kinases (RTKs), including c-MET, EGFR, VEGFR, PDGFR, HER2/HER3, and FLT3. Anticancer therapies utilizing small-molecule inhibitors against RTKs are important treatment strategies for advanced-stage human malignancies.^1–4^ However, acquired drug resistance remains a serious concern, limiting the long-term efficacy of these inhibitors. Thus, urgent measures are needed to identify novel therapeutic targets to prevent resistance and limit tumor progression. AXL, a member of the TAM (TYRO3, AXL, and MERTK) family of RTKs, has emerged as a promising target. The name AXL is derived from the Greek word *anexelekto*, meaning uncontrolled, reflecting its involvement in cancer progression. TAM RTKs are recognized for their role in various biological functions, including promoting apoptotic cell phagocytosis, maintaining vascular integrity and modulating immune responses.^5–8^ In addition, they serve as phosphatidylserine-mediated virus entry-enhancing receptors (PVEERs), facilitating the infiltration of enveloped viruses into human cells.^9–11^ Structurally, all three TAM share common features, where ligand binding induces homodimerization, leading to autophosphorylation and kinase activation. This activation triggers several downstream signaling pathways involved in cancer onset and progression.^12–14^ Unlike many RTKs, TAM receptors are rarely mutated. Instead, their dysregulation is typically due to overexpression caused by gene amplification or stimulation by the tumor microenvironment. This makes TAM receptors, particularly AXL, attractive therapeutic targets with a lower risk of developing drug resistance through mutations.^15^ AXL has gained attention in cancer therapy due to its frequent overexpression in various malignancies including renal,^16,17^ lung,^18^ breast,^19^ prostate,^20^ colon,^21,22^ esophageal,^23^ and head and neck cancers.^24,25^ Its role in therapeutic resistance has spurred several clinical trials.^26^ Notably, the selective AXL inhibitor bemcentinib (BGB324/R428) recently received fast-track designation by the US FDA in combination with a PD-1/PD-L1 agent, highlighting its growing importance in cancer research. AXL plays critical role in cancer cell proliferation, stemness, migration, invasion, angiogenesis, therapeutic resistance and poor prognosis.^27,28^ For instance, in prostate cancer, AXL regulates cell proliferation through the Akt/NF-kβ pathway.^20^ In breast cancer, it maintains acquired stemness by co-opting epithelial plasticity programs within mammary stem cells.^29^ Beyond cancer cells, AXL promotes angiogenesis by supporting endothelial cell function, facilitating metastasis, as seen in hepatocellular carcinoma (HCC) through activation of the PI3K/Akt/SOX2/DKK-1 network.^30^ In head and neck cancer, AXL derives resistance to cetuximab by upregulating the HER3 ligand neuregulin1.^31^ Similarly, in non-small-cell lung cancer (NSCLC), AXL enhances MYC transcription, which disrupts purine metabolism and increases the likelihood of drug-resistant mutations.^32^ In addition, patients with pancreatic ductal adenocarcinoma (PDA),^33^ clear cell renal cell carcinoma (ccRCC),^34^ and cholangiocarcinoma (CCA)^35^ often face poor prognosis due to dysregulation of the growth arrest-specific protein 6 (GAS6)/AXL signaling pathway. These findings collectively suggest that AXL could offer significant potential for treating various cancers and preventing therapeutic resistance.

In this review, we explore recent studies on structural and functional characteristics of AXL, its regulatory mechanisms, and the crosstalk with other RTKs. We also examine preclinical and clinical evidences of AXL’s role in therapeutic resistance. Finally, we discuss the translational relevance of AXL-targeted therapies in enhancing the patient outcomes by reducing resistance and promoting tumor regression.

## Structure, signaling, regulation, and functions of AXL

### Structure of AXL receptor

The AXL gene is located at locus 19q13.2, near the BCL3 proto-oncogene. It consists of 20 exons encoding an 894 amino acid multidomain protein.^26^ AXL is a 140 kDa single-pass transmembrane receptor, comprising extracellular, transmembrane and intracellular domains. The extracellular domain of AXL contains two immunoglobulin (Ig)-like repeats and two fibronectin type III (FNIII)-like repeats, structurally resembling neural cell adhesion molecules (NCAMs).^36^ AXL binds to its ligand GAS6, primarily through the Ig motifs, with regulation modulated by the FNIII.^37^ The intracellular domain is crucial for autophosphorylation and subsequent kinase activity.^38^ Upon GAS6 binding, AXL undergoes dimerization and is activated through phosphorylation at specific tyrosine residues. This phosphorylation event is essential for the recruitment of key adaptor and effector molecules, initiating downstream signaling pathways from AXL.^39^

### AXL ligands

**Fig. 1:**
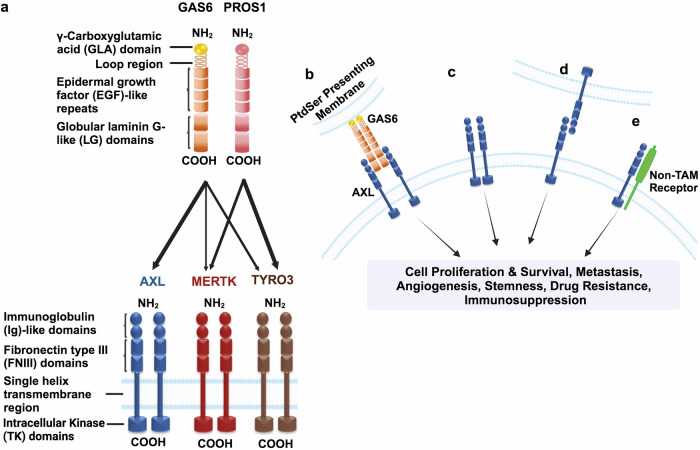
Structure and activation mechanisms of the TYRO3, AXL, and MERTK (TAM) receptors. **a** The TAM receptors and ligands share closely related structures. The structure of growth arrest-specific protein 6 (GAS6) and vitamin K-dependent protein S (PROS1) comprises a γ-carboxyglutamic acid (GLA) domain, a loop region, four epidermal growth factor-like (EGF-like) repeats, and two C-terminal globular laminin G-like (LG) domains. TAM receptors consist of two immunoglobulin-like (Ig) domains, two fibronectin III (FNIII) repeat domains, and a kinase domain. GAS6 binds to all three receptors, while PROS1 binds to MERTK and TYRO3, but not to AXL. The decreasing width of the arrows indicates a reduction in receptor activation strength by the ligands, reflecting their relative affinities. **b** Ligand-Dependent Activation: Binding of GAS6 to AXL leads to receptor activation. Optimal activation requires PtdSer (phosphatidylserine) on the opposed cell membrane, which binds to the GLA domain of the ligand. **c** Ligand-Independent Activation: Various factors in the tumor microenvironment can activate AXL in a ligand-independent manner. **d** Homophilic Interaction: Activation can occur through interaction between AXL monomers on neighboring cells. **e** Heterophilic Activation: AXL can also be activated heterophilically with non-TAM receptors. Activation of AXL through these mechanisms results in autophosphorylation of intracellular tyrosine residues, initiating signaling pathways that promote tumor proliferation, invasion, migration, angiogenesis, drug resistance, and immune evasion

Initially believed to be orphan receptors, the TAM family of receptors are now known to have several ligands,^40^ including GAS6, protein S (Pros1), Tubby, Tubby-like protein 1 (Tulp-1), and galectin-3.^41^ GAS6 and protein S are vitamin K-dependent secretory proteins that bind to the extracellular domains of AXL, TYRO3, and MER, triggering dimerization and phosphorylation within the cytoplasmic domain, leading to receptor activation.^42,43^ Among these, ligands, GAS6 and Tulp-1 are recognized as universal ligands for all the TAM receptors, binding to each member of the TAM family.^44^ The full activation of these receptors, particularly in both healthy tissues and the cancer microenvironment, depends on their interactions with ligands and phosphatidylserine (PtdSer). GAS6 binds to PtdSer present on the cell membrane, forming an extracellular lipid-protein complex. This complex is critical for initiating TAM receptor dimerization and activating of the AXL signaling pathway, crucial for cancer cell survival and proliferation (Fig. 1).^45^

This interaction between GAS6 and AXL, mediated by Ig motifs and FNIII, is unique. It prevents direct AXL/AXL or GAS6/GAS6 interactions, influencing processes such as epithelial-to-mesenchymal transition (EMT), a key event in cancer progression.^46^

### Activation and regulation of AXL signaling

The activation of the AXL receptor occurs through several mechanisms: ligand-dependent (GAS6-driven dimerization), ligand-independent (atypical) activation, monomer interactions with neighboring cells, and heteromeric dimerization with non-TAM receptors.^42,47,48^

#### Mechanisms of AXL activation

##### GAS6-dependent AXL activation

AXL binds to GAS6 in a 1:1 ratio, forming a complex that undergoes dimerization, activating downstream signaling pathways such as phosphatidylinositol 3-kinase/Akt kinase (PI3K/Akt) and mitogen-activated protein kinase/extracellular signal-regulated kinase (MAPK/ERK). These pathways are involved in promoting cancer cell growth and metastasis.^14,37,49,50^

##### GAS6-independent AXL activation

Beyond the canonical GAS6-driven mechanism, AXL can be activated in the absence of GAS6 through crosstalk with other receptors like c-MET or EGFR,^51^ self-dimerization,^52^ or as a response to oxidative stress.^53^ These alternative mechanisms further drive cancer progression.

#### AXL regulation

AXL signaling is tightly regulated through a range of complex mechanisms, including epigenetic alterations, transcriptional, translational, and posttranslational modifications.^26^ Several studies have demonstrated that AXL gene expression can be modulated by promoter methylation,^54^ as well as by various transcription factors (HIF-α, AP-1, YAP1/TEAD, CREB, MZF1, and SP-1)^55–59^. In addition, interactions with miRNAs also play a role in the regulation of AXL expression.^60^

Posttranslational modifications, including glycosylation, phosphorylation, proteolytic cleavage, and ubiquitination, further influence the functionality of AXL in cancer progression.^61–63^ Beyond these mechanisms, studies have identified the role of long non-coding RNA (LncRNAs) (DANCR, and XIST), which act as endogenous RNAs that compete with miRNAs (miR-33a-5p, and miR-93-5p) to regulate AXL signaling at the post-transcriptional level in various malignancies.^64–66^ In addition to these regulatory mechanisms, transcriptomic sequencing of primary lung adenocarcinomas revealed the AXL-MBIP (MAP3K12 binding inhibitory protein 1) fusion gene as another potential mechanism for AXL activation.^67^ These findings expand the understanding of AXL’s regulatory landscape and could provide new avenues for the development of targeted cancer therapies.

### Biological functions of AXL and its role in cancer progression

**Fig. 2:**
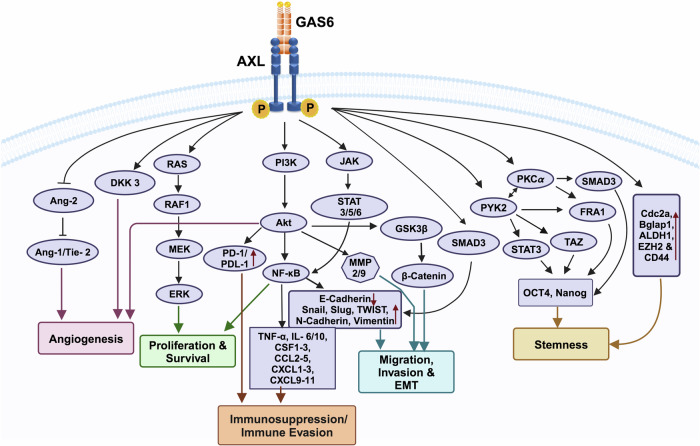
AXL regulates multiple biological processes in cancer. AXL is pivotal in regulating several key biological processes in cancer. Upon binding to its ligand, GAS6, AXL becomes activated, triggering downstream signaling pathways that promote cancer cell proliferation, survival, migration, invasion, epithelial-to-mesenchymal transition (EMT), angiogenesis, stem cell maintenance, and immune suppression or evasion

AXL overexpression has been linked to several key biological processes that drive cancer development and metastasis. These functions, outlined below, highlight the crucial role of AXL in promoting tumor progression (Fig. 2).

#### AXL in cell proliferation and survival

AXL signaling plays a pivotal role in promoting cell proliferation through various downstream pathways, including PI3K/Akt, JAK/STAT, NF-κβ, and RAS/RAF/MEK/ERK.^68,69^ In addition, AXL enhances cell survival by regulating NF-κβ nuclear transport, which leads to increased expression of anti-apoptotic markers such as survivin and BCL-2, while simultaneously reducing the expression of pro-apoptotic markers like caspase 3 and BAD.^70^ AXL inhibition has been shown to lower the levels of anti-apoptotic proteins, such as MCL-1 and BCL-2, while upregulating apoptosis-related proteins like Bim, particularly in chronic lymphocytic leukemia (CLL).^71^ Blocking the GAS6/AXL axis is critical for suppressing tumor growth in various cancers, including renal cell carcinoma (RCC), prostate cancer, lung adenocarcinoma, and colorectal cancer.^72^ For example, a study by Yu et al. identified AXL as a key survival factor for RCC cells, where it is overexpressed in human RCC tissues and cell lines. Targeting AXL using the monoclonal antibody hMAb173 induces apoptosis in both ex vivo and in vivo models.^73^ These findings suggest that AXL plays a significant role in cancer cell proliferation and survival, making it a promising therapeutic target to inhibit cancer growth and enhance apoptosis in a range of malignancies.

#### AXL in cell migration and invasion

AXL overexpression is strongly linked to increased cell migration and invasion across various cancer types. AXL activity has been associated with the upregulation of GTP-binding proteins, such as Rho and Rac, which are key facilitators of cell migration.^74^ Additionally, AXL activation promotes the expression of important factors such as Akt and MMP9, primarily through NF-κβ and Brg-1 signaling pathways.^70,75^ In cancers, such as oral squamous cell carcinoma (OSCC) and ovarian tumors, AXL activation stimulates the PI3K/Akt pathway that mediates cancer cell invasion through increased expression of proteolytic enzymes like MMP2 and MMP9. In hepatocellular carcinoma, studies have shown that YAP1 knockdown results in reduced AXL activity, leading to decreased cell invasion. This emphasizes the significant role of AXL in invasion and migration processes.^76^ Furthermore, AXL regulates genes associated with metastasis (e.g., somatostatin receptor 2 (*SSTR2)*, fms-related receptor tyrosine kinase 4 (*FLT4*), matrix metalloproteinase 10 (*MMP10*), kisspeptin-1 (*KISS1*), collagen type IV alpha 2 chain (*COL4A2*), RAR-related orphan receptor B (*RORB*)) and is linked to the expression of stem cell markers.^77^ It also facilitates the migration and invasion of breast cancer stem cells. Notably, AXL is involved in the production of cathepsin B, a facilitator of invasion, and the peripheral distribution of lysosomes in esophageal cancer cell lines.^78^ These findings collectively suggest that AXL could be a promising target to inhibit cancer invasion and migration, thereby disrupting tumor progression.

#### AXL in epithelial-to-mesenchymal transition (EMT)

Recent studies have demonstrated that AXL plays a role in promoting EMT. Activation of AXL initiates a reversible transition from an epithelial to a mesenchymal phenotype, enhancing cell invasiveness and metastatic potential.^79^ Several growth factors, including TGF-β, PDGF, and HGF as well as transcription factors such as Slug, Snail, ZEB1, and TWIST1, mediate EMT in various cancers.^77,80^ AXL has been identified as a phenotypical marker in Non-small-cell lung cancer (NSCLC) cell lines and breast cancer stem cells.^79,81^ Elevated levels of AXL are crucial for the downregulation of certain pro-epithelial factors such as E-cadherin, while simultaneously upregulating mesenchymal markers like N-cadherin, Slug, Snail, α-catenin, α-SMA, and vimentin.^82,83^

In addition to downregulating pro-epithelial factors and inducing mesenchymal markers, AXL enhances the transcriptional expression of EMT regulators Slug and N-cadherin by activating PI3K and NF-κβ pathways.^77,84^ Overexpression of AXL in primary breast cancers is associated with reduced patient survival. Experimental breast cancer models have shown that knocking down AXL inhibits the metastasis of highly invasive breast carcinoma cells to lymph nodes and other organs, leading to improved overall survival.^81^ Similarly, TGF-β promotes AXL expression in HER2-positive breast cancer tissues, contributing to their invasive and metastatic properties.^85^ In summary, AXL plays a dual role in promoting EMT across various cancers. It functions as an upstream signaling molecule that drives EMT and maintains cells in a mesenchymal state independently of GAS6. This mesenchymal state can be reversed by downregulating AXL, restoring an epithelial-like morphology.

EMT is also involved in the formation of circulating tumor cells (CTCs), which drive metastatic relapse and are key components of liquid biopsies.^86^ A study by de Miguel-Pérez et al. suggested that assessing both tissue AXL expression and CTCs could improve predictions of patient outcome and tailor post-surgical therapy for resected lung adenocarcinomas.^87^ Currently, CellSearch is the only FDA-cleared platform for CTC enumeration, demonstrating clinical value across various malignancies.^88–90^ However, the limited expression of epithelial cell adhesion molecule (EPCAM) in CTCs has restricted their utility in advanced tumors.^91–93^ Microcavity array technology, utilizing mesenchymal markers, has proven effective in identifying AXL-expressing CTCs in advanced NSCLC patients.^94^ Furthermore, Bardol et al. provided the first evidence of CellSearch® detecting AXL^+^ CTCs in metastatic breast cancer, paving the way for future studies to explore anti-AXL treatments.^95^

#### AXL in angiogenesis

TAM receptors, including AXL, are crucial not only for cancer cell proliferation and survival but also for vessel integrity and angiogenesis. They modulate various growth factors such as VEGF, FGF, and PDGF, which are essential for these processes.^96,97^ AXL is expressed in endothelial and vascular smooth muscle cells, where it supports the stabilization and survival of endothelial cells and contributes to tissue remodeling, wound healing, and vessel integrity.^98^ During neoplasm formation, both AXL and its ligand GAS6 are often overexpressed. They regulate the expression of matrix proteins, promoting endothelial cell proliferation, migration, and survival.^99^ AXL has been implicated in stimulating growth and tube formation in human umbilical vein endothelial cells (HUVEC), through the VEGF-A-mediated activation of the PI3K/Akt pathway, which is crucial for tumor angiogenesis.^100,101^ Knockdown studies of AXL and GAS6 have demonstrated reduced endothelial tube formation, attributed to decreased expression of pro-angiogenic proteins such as Dickkopf-related protein 3 (DKK3) and angiopoietin-2 (Ang-2),^27^ which are key mediators of tumor angiogenesis. Inhibitors of AXL, when used in conjunction with antiangiogenic agents, have shown a significant reduction in vessel or tube formation in renal carcinoma xenografts.^102^ In addition, the GAS6/AXL/S100A10 axis has been found to promote plasmin generation, endothelial cell migration, and angiogenesis in clear cell renal cell carcinoma (ccRCC) cells, with AXL expression being associated with resistance to antiangiogenic therapies.^102^

#### AXL in stemness

AXL is a key driver of stemness in cancer cells, facilitating self-renewal and contributing to the long-term maintenance of tumors. Its expression corelates with various cancer stem cell markers, including cell division cycle 2a (Cdc2a), bone gamma-carboxyglutamate protein 1 (Bglap1), aldehyde dehydrogenase 1 (ALDH1), enhancer of zeste homolog 2 (EZH2) and cluster of differentiation 44 (CD44).^70,103,104^ Increased levels of these stemness markers enhance resistance to chemotherapy in squamous cell carcinoma and promote self-renewal activities in human mammary epithelial cells.^105^ AXL maintains breast cancer stem cells (BCSCs) and epithelial plasticity through a positive feedback loop. Inhibiting AXL with inhibitors such as MP-470 or amuvatinib can effectively reverse the EMT process in BCSCs by downregulating the NF-κβ pathway. AXL expression is linked to the regulation of stem cell genes, metastatic genes, and enhanced tumor progression through increased cellular invasion and migration.^77^ Specifically, the AXL-PYK2 (proline-rich tyrosine kinase 2)-PKCα (protein kinase C alpha) signaling axis has been shown to induce stemness in triple-negative breast cancer (TNBC). In TNBC, the expression of AXL, PYK2, and PKCα correlates with stemness in breast cancer patients. These molecules activate signal transducer and activator of transcription (STAT3), SMAD family member 3 (SMAD3), transcriptional coactivator with PDZ-binding motif (TAZ), and Fos-related antigen-1 (FRA1), along with pluripotent stem cell transcription factors Nanog and Oct4.^106^

In cutaneous squamous cell carcinoma (SCC), AXL contributes to cell invasiveness and stemness by modulating Wnt and TGF-β signaling pathways and disrupting cell-to-cell adhesion.^103^ Furthermore, suppressing AXL through inhibitors or shRNA extends the survival of chronic myeloid leukemia (CML) mice and reduces the growth of leukemia stem cells (LSCs). The GAS6/AXL complex has been shown to stabilize β-catenin in human CML CD4+ cells, presenting a potential target for eliminating CML LSCs.^107^ Therefore, targeting AXL or its signaling pathways may enhance clinical response to anticancer therapies by reducing stemness, recurrence and metastasis.

#### AXL in immunosuppression

In recent years, significant progress has been made in cancer immunotherapy; however, acquired resistance remains a major clinical challenge. AXL has emerged as a key player in the development of tumor immune tolerance.^26^ AXL induces immune evasion by upregulating BCL-2 and Twist, suppressing inflammatory signaling, and reducing the production of pro-inflammatory cytokines and chemokines such as interleukin 1 alpha (IL-1 α), interleukin 6 (IL-6), tumor necrosis factor-alpha (TNF-α), C-X-C motif chemokine ligand 1, 2 and 5 (CXCL1, CXCL2, CXCL5), colony-stimulating factor 1-3 (CSF1-3) and C-C motif chemokine ligand 2-5 (CCL2-5).^108^ It also contributes to radio-resistant and checkpoint immune checkpoint resistance by blocking MHC-I antigen presentation and promoting the production of myeloid-supporting chemokines and cytokines, which trigger an adverse immune response.^109,110^ AXL confers therapeutic resistance by activating the PI3K/Akt pathway and increasing the transcription of programmed cell death ligand 1 (PD-L1) in head and neck cancer cells.^111,112^ Overexpression of AXL has been linked to both intrinsic and acquired resistance to immune checkpoint blockade (ICB) in real cell carcinoma (RCC). A strong correlation exists between AXL and PD-L1 expression, with high levels of AXL in tumor cells associated with lower response rates and shorter progression-free survival following anti-PD-1 treatment. Furthermore, patients with tumors that display both high PD-L1 and AXL expression have the poorest overall survival and a reduced antitumor immune response.^113^

Knockout studies of AXL have demonstrated reduced tumor growth and increased sensitivity to immunotherapy due to the infiltration of CD8 + T cells in a murine model of breast cancer.^114,115^ This indicates that AXL plays a multifaceted role in immunosuppression by regulating the secretion of immunosuppressive cytokines and mediators.^116^ AXL-mediated remodeling of the tumor microenvironment (TME) leads to the downregulation of immune effector cells, such as natural killer (NK) cells, T cells, and dendritic cells, while promoting the recruitment of immunosuppressive cells such as regulatory T cells (Tregs), tumor-related macrophages, and myeloid-derived suppressor cells (MDSCs).^117^

In addition, AXL signaling results in a reduction of cytotoxic T cells at effector sites and induces the transformation of macrophages from M1 to M2 polarization, ultimately facilitating tumor evasion and a poor prognosis.^118^ Therefore, inhibiting AXL can remodel the TME, enhance immune response and improve the therapeutic potential of immune checkpoint blockade inhibitors.

#### AXL in viral infections and inflammatory responses

Beyond its role in immune regulation, AXL has been implicated in the progression and severity of various viral infections, including Ebola virus (EBOV), severe acute respiratory syndrome coronavirus 2 (SARS-CoV-2), and Zika virus (ZIKV).^119–121^ Its interactions with these viruses highlight its dual role in facilitating viral entry and modifying inflammatory responses, establishing a connection between infectious diseases and cancer progression.

Using a series of AXL mutants, Shimojima et al. demonstrated that EBOV particles activate AXL through cell surface adhesion and interaction with its immunoglobulin domains, enabling viral entry by endocytosis.^120^ Similarly, AXL has been identified as a potential receptor for SARS-CoV-2, interacting directly with the spike protein’s N-terminal to facilitate viral attachment and entry into pulmonary epithelial cells.^122^ During Zika virus infection, AXL enhances viral entry in glial cells through GAS6 bridging and impacts innate immune responses.^119^ Studies involving Zika virus strain MR-766 in human glioblastoma cell lines revealed that AXL is essential for viral entry, though the exact mechanisms remain unclear and needs further investigations.^123^ Moreover, AXL was recently identified as a co-receptor for parvovirus B19 in human erythroid progenitors, further emphasizing its role in viral pathogenesis.^124^ These findings suggest that targeting AXL could be a promising strategy for future antiviral treatments.

In addition to facilitating viral entry, AXL plays a crucial role in regulating inflammatory responses. Activation of AXL by its ligand, GAS6, triggers a signaling cascade that suppresses innate immune defenses, allowing viruses to evade detection. This occurs through the upregulation of suppressors of cytokine signaling (SOCS), which in turn inhibits toll-like receptor (TLR) pathways essential for a robust immune response.^125^ While this immunomodulatory function can reduce tissue damage during infections, it also creates a vulnerability to malignancy. Tumors often exploit these inflammatory pathways to escape immune surveillance, and elevated AXL expression can impair the function of natural killer (NK) cells and macrophages, weakening the antitumor immune response. For instance, the human papillomavirus type 16E6 (HPV16E6) oncoprotein has been shown to upregulate AXL expression in cervical cancer via the MAGI-2/PTEN/Akt/MZF1 network, increasing tumor invasiveness and promoting immune evasion by reducing responsiveness to NK cells-mediated lysis.^126^

Thus, AXL serves as a critical link between viral infections, inflammatory responses, and cancer progression.^127^ By facilitating viral entry and modulating the host’s immune response, AXL contributes to the pathogenesis of both infectious diseases and cancer metastasis. These insights position AXL as a potential therapeutic target in both virology and oncology, where its inhibition could enhance antitumor immunity and improve clinical outcomes.

However, studies suggest that AXL inhibition may also create a tumor-promoting environment by enhancing pro-inflammatory cytokine production and impairing the clearance of apoptotic neutrophils.^128^ This is particularly relevant in comorbid conditions like inflammatory arthritis, where AXL, regulated by miR-34a, is essential for controlling dendritic cell (CD1c+ DC) activation and the generation of pro-inflammatory cytokines such as TNF, IL-6, IL-1β, and IL-23.^129^ Moreover, AXL deficiency has been observed to exacerbate pulmonary arterial hypertension (PAH) via the bone morphogenetic protein receptor 2 (BMPR2) pathway, suggesting a protective role for the GAS6/AXL axis in PAH.^130^ Therefore, AXL’s paradoxical roles as both a proto-oncogene and an immunological protector highlight its complexity in cancer and related comorbidities. Blocking AXL could interfere with its beneficial immunoregulatory effects, potentially exacerbating inflammation or immune dysregulation in comorbid diseases. Thus, a thorough investigation of AXL’s context-dependent effects is necessary to develop effective therapies.

Future research should focus on clarifying AXL’s roles in viral pathogenesis, tumor biology, and related comorbidities, paving the way for innovative treatment strategies. Nonetheless, the unpredictable mutations in viral genomes present a significant challenge for therapeutic development, necessitating further investigation.

## Targeted therapy toward AXL

The studies discussed above confirm the pleiotropic effects of AXL on cancer growth, metastasis, and drug resistance, establishing it as a promising target for anticancer therapies. AXL inhibitors have shown significant benefits both in *vitro and* in vivo by promoting apoptosis and reducing cell proliferation and migration.^131^ Targeting AXL can also enhance the therapeutic efficacy of chemotherapy^132^, and various small-molecule inhibitors that target EGFR, HER2, PARP, PI3K, and VEGF-A.^133–137^ Currently, different types of AXL inhibitors are in various stages of development including small-molecule tyrosine kinase inhibitors (TKIs) that block AXL kinase activity, anti-AXL antibodies against AXL receptors, soluble AXL decoy receptors for neutralizing GAS6, nucleotide aptamers that bind to and inhibit AXL, and natural compounds as AXL inhibitors.^70,138^ In this review, we primarily focus on small-molecule AXL inhibitors and anti-AXL antibodies that currently being investigated in preclinical and clinical studies for the treatment of various human malignancies (Table 1).

**Table 1:** Targeted therapies against AXL

Small-molecule AXL TKI	Target(s)	Mechanism(s)		Cancer type	Monotherapy/combination			Clinical trial no./phase^a^	Status^a^			Adverse events
Selective AXL Inhibitors												
Bemcentinib/BGB324/R428	AXL	Inhibition of Akt and MAPK pathways, upregulate PUMA and downregulate Bcl-2		NSCLC	+ Docetaxel			NCT02922777- I	Completed			Neutropenia, diarrhea, nausea, vomiting, QTc prolongation, transaminase increase, asthenia, and fatigue
				AML or MDS	Monotherapy in AML or MDS/+ cytarabine or decitabine in AML			NCT02488408- Ib/II	Unknown status			
				NSCLC	+ Erlotinib			NCT02424617- I/II	Completed			
				TNBC or TN-IBC	+ Pembrolizumab			NCT03184558- II	Terminated			
				MDS patients failing standard-of-care therapy	Monotherapy			NCT03824080- II	Completed			
				Advanced NSCLC	+ Pembrolizumab			NCT03184571- II	Completed			
				Malignant mesothelioma	+ Pembrolizumab			NCT03654833- II	Active, not recruiting			
				Metastatic melanoma	+ Pembrolizumab or Dabrafenib/Trametinib			NCT02872259- Ib/II	Completed			
				Recurrent glioblastoma undergoing surgery	Monotherapy			NCT03965494- I	Terminated			
				Metastatic pancreatic cancer	+ Chemotherapy (nab-paclitaxel/gemcitabine)			NCT03649321- I/II	Terminated			
				Untreated non-squamous NSCLC	+ Pembrolizumab/carboplatin/pemetrexed			NCT05469178- Ib/IIa	Recruiting			
TP-0903/Dubermatinib	AXL	Inhibit Aurora B activation to induce G2/M arrest; Induction of apoptosis by suppressing Mcl-1, Bcl-2 and XIAP; upregulation of BIM		Advanced solid tumors	Monotherapy			NCT02729298- I	Completed			Thrombocytopenia, anemia, nausea, syncope, and vomiting
				FLT3 mutated AML	TP-0903 alone/+azacitidine			NCT04518345- Ib/II	Completed			
				AML	Monotherapy			NCT03013998- Ib/II	Recruiting			
				Previously treated CLL	Monotherapy/+ ibrutinib			NCT03572634-I/II	Terminated			
DS-1205	AXL	Inhibit GAS6-mediated cell migration		Metastatic or unresectable EGFR-mutant NSCLC	+ Gefitinib			NCT03599518-I	Terminated			Combination with gefitinib may induce elevation of liver enzymes, vomiting, diarrhea, and fatigue
				Metastatic or unresectable EGFR-mutant NSCLC	+ Osimertinib			NCT03255083-I	Terminated			
AVB-S6-500/Batiraxcept	AXL decoy receptor	Inhibit GAS6-induced AXL or Src phosphorylation		Platinum-resistant recurrent ovarian cancer		+ Pegylated liposomal doxorubicin or paclitaxel		NCT03639246- I		Completed		Avelumab in combination with AVB-S6-500 may result in UTI, hyponatremia, elevated creatinine, anemia, thrombocytopenia, hematuria, anorexia, and sepsis
				Platinum-resistant recurrent ovarian, fallopian tube or primary peritoneal cancer		+ Durvalumab (MEDI4736)		NCT04019288- I/II		Active, not recruiting		
				Platinum-resistant recurrent ovarian cancer		+ Paclitaxel vs placebo and paclitaxel		NCT04729608- III		Terminated		
				Advanced or metastatic ccRCC		AVB-S6-500 alone/+ cabozantinib/+ cabozantinib and nivolumab		NCT04300140- Ib/II		Terminated		
				Advanced pancreatic adenocarcinoma		+ Nab-paclitaxel and gemcitabine		NCT04983407- Ib/II		Terminated		
				Advanced urothelial carcinoma		+ Avelumab		NCT04004442- I		Active, not recruiting		
Multitargeted AXL inhibitors												
BMS777607	AXL, RON, c-MET, TYRO3, MER		Inhibition of HGF-induced c-MET-phosphorylation and downregulate MEK-ERK and PI3K/Akt pathways	Advanced or metastatic solid tumors			Monotherapy	NCT01721148-I			Completed	Anemia, nausea, Constipation
				Advanced or metastatic solid tumors			Monotherapy	NCT00605618- I/II			Completed	
Cabozantinib/BMS-907351	AXL, VEGFR2, c-MET, RET, TIE2, c-KIT, Flt3, ROS1		Decrease AXL phosphorylation and TGF-β-induced E-cadherin expression	NSCLC			With or without erlotinib	NCT00596648- Ib/II			Completed	Fatigue, diarrhea, hypertension, palmar-plantar erythrodysesthesia syndrome, fistula, abdominal and pelvic abscess
				Previously treated metastatic NSCLC			+ Erlotinib	NCT01866410- II			Completed	
				Stage IV NSCLC			Monotherapy/+ erlotinib	NCT01708954- II			Active, not recruiting	
				KRAS wild-type metastatic colorectal cancer			Monotherapy in MET amplified metastatic colorectal cancer/+ panitumumab	NCT02008383- I			Completed	
				Locally advanced or metastatic solid tumors			Cabozantinib alone / + atezolizumab	NCT03170960- I/II			Active, not recruiting	
				Metastatic castration-resistant prostate cancer			+ Atezolizumab vs second novel hormonal therapy	NCT04446117- III			Active, not recruiting	
				Advanced HCC with no previous systemic anticancer therapy			+ Atezolizumab vs sorafenib	NCT03755791- III			Active, not recruiting	
				Metastatic NSCLC previously treated with anti-PD-L1/ PD-1 Antibody and platinum- chemotherapy			+ Atezolizumab vs docetaxel monotherapy	NCT04471428- III			Active, not recruiting	
				Advanced RCC after immune checkpoint inhibitor treatment			+ Atezolizumab vs cabozantinib alone	NCT04338269- III			Active, not recruiting	
				Previously untreated advanced or metastatic RCC			+ Nivolumab vs Sunitinib	NCT03141177- III			Active, not recruiting	
				Non-clear cell RCC			+ Nivolumab	NCT03635892- II			Active, not recruiting	
				Previously untreated advanced or metastatic RCC			+ Nivolumab and ipilimumab vs nivolumab and ipilimumab with matched placebo	NCT03937219- III			Active, not recruiting	
				NSCLC			With or without erlotinib	NCT00596648- Ib/II			Completed	
				Advanced kidney cancer			Ipilimumab and nivolumab followed by nivolumab alone vs nivolumab with cabozantinib	NCT03793166- III			Active, not recruiting	
				Recurrent stage IV NSCLC			+ Nivolumab or + nivolumab and ipilimumab vs nivolumab alone	NCT03468985- II			Active, not recruiting	
				Advanced liver cancer			Nivolumab alone/ nivolumab and sorafenib/ nivolumab and ipilimumab/ + nivolumab/ + nivolumab and ipilimumab	NCT01658878-I/II			Active, not recruiting	
				Radioiodine-refractory differentiated thyroid cancer progressed after prior VEGFR -targeted therapy			Cabozantinib vs placebo	NCT03690388-III			Active, not recruiting	
				Advanced pancreatic neuroendocrine and carcinoid tumors			Cabozantinib vs Placebo	NCT03375320-III			Active, not recruiting	
				Hepatocellular carcinoma patients received prior sorafenib			Cabozantinib vs Placebo	NCT01908426- III			Completed	
				Metastatic RCC			Cabozantinib vs Everolimus	NCT01865747- III			Completed	
Bosutinib/SKI-606	AXL, Src kinase, BCR-ABL		Inhibits autophosphorylation of AXL with downregulation of slug and inhibits PI3K/Akt/mTOR pathways	Recurrent glioblastoma			Monotherapy	NCT01331291- II			Completed	Diarrhea, rash, liver enzyme elevations
				Advanced breast cancer			Monotherapy	NCT00319254- II			Completed	
				Advanced malignant solid tumors			Monotherapy	NCT01001936- I			Completed	
				Philadelphia chromosome +ve CML previously treated with one or more TKIs			Monotherapy	NCT02228382- IV			Terminated	
				CML-CP previously treated with 2 or more TKIs			ABL001 vs Bosutinib	NCT03106779- III			Active, not recruiting	
				Selected metastatic solid tumors			+ Pemetrexed	NCT03023319- I			Completed	
				HR + HER2- advanced breast cancer refractory to CDK4/ 6 inhibitor			+ Palbocicilib and fulvestrant	NCT03854903-I			Active, not recruiting	
Crizotinib/PF-02341066	AXL, ALK, c-MET, RON		Inhibits phosphorylation of target receptors	ALK+ advanced NSCLC			Brigatinib vs Crizotinib	NCT02737501- III			Completed	Abdominal pain, headache, pain in extremity, nausea, pyrexia
				High-risk uveal melanoma following definitive therapy			Monotherapy	NCT02223819- II			Completed	
				c-MET +ve gastric adenocarcinoma			Monotherapy	NCT02435108- II			Completed	
				mCRPC			+ Enzalutamide	NCT02207504- I			Completed	
Sunitinib/SU11248	AXL, c-KIT, FLT3, PDGFR, VEGFR2		Inhibits ligand-mediated VEGFR2, PDGFR-β phosphorylation	Metastatic RCC			Monotherapy	NCT00706706- IV			Completed	Diarrhea, fatigue, hypertension, hematologic adverse events and palmar-plantar erythrodysesthesia syndrome
				Refractory solid tumors			Monotherapy	NCT02691793- IV			Completed	
				High-risk non-muscle invasive lower urinary tract urothelial carcinoma			Monotherapy	NCT00794950- II			Completed	
				Advanced cholangiocarcinoma			Monotherapy	NCT01718327- II			Completed	
				Advanced well-differentiated pancreatic neuroendocrine tumors			Monotherapy	NCT01525550- IV			Completed	
				Imatinib resistant or intolerant malignant GIST			Monotherapy	NCT00793871- IV			Completed	
				Locally advanced or recurrent soft tissue sarcoma			Preoperative sunitinib and radiation	NCT01498835- I			Completed	
				Recurrent ovarian clear cell carcinoma			Monotherapy	NCT01824615- II			Completed	
				NSCLC patients with brain metastases			Monotherapy	NCT00372775-II			Completed	
				Metastatic or recurrent thymic carcinoma			Monotherapy	NCT02623127- II			Completed	
Sitravatinib/MGCD516	AXL, c-KIT, VEGFR3 PDGFR, c-MET, RET, MER, FLT3		Immune-modulation of tumors by reducing immunosuppressive myeloid cells and increase CD4^+^ & CD^+^8 T cells	Metastatic, pre-treated TNBC			Monotherapy	NCT04123704- II			Terminated	Diarrhea, fatigue, rash, nausea
				ccRCC			+ Nivolumab	NCT03680521- II			Completed	
				Advanced solid tumor malignancies			Monotherapy	NCT02219711- I/Ib			Completed	
				Advanced or metastatic kidney cancer progressed on prior VEGF-targeted therapy			+ Nivolumab	NCT03015740- I/II			Completed	
				Locally recurrent or metastatic TNBC			+ Tislelizumab/ + tislelizumab and nab-paclitaxel	NCT04734262- II			Active, not recruiting	
				Advanced solid tumors			+ Tislelizumab	NCT03666143- Ib			Completed	
				NSCLC			+ Nivolumab	NCT02954991- II			Terminated	
				Advanced or metastatic ccRCC or other solid malignancies			+ Nivolumab and ipilimumab	NCT04518046- I/Ib			Completed	
				Advanced or metastatic urothelial carcinoma			+ PD-(L)1 checkpoint inhibitors regimens (nivolumab/ pembrolizumab and enfortumab vedotin)	NCT03606174- II			Terminated	
				Advanced NSCLC			+ Nivolumab vs Docetaxel	NCT03906071- III			Active, not recruiting	
				Unresectable locally advanced or metastatic HCC or GC/GEJC			Monotherapy/ + tislelizumab	NCT03941873- I/II			Completed	
				Locally advanced or metastatic NSCLC			+ Tislelizumab vs Docetaxel	NCT04921358- III			Terminated	
				Advanced biliary tract cancer			+ Tislelizumab	NCT04727996- II			Active, not recruiting	
				Advanced treatment-naïve PD-L1+ non-squamous NSCLC			+ Pembrolizumab	NCT04925986- II			Terminated	
				Advanced or metastatic malignancies			Monotherapy/ + other anticancer therapies (nivolumab, pembrolizumab, enfortumab vedotin, ipilimumab)	NCT04887870- II/III			Active, not recruiting	
S49076	AXL/MET, c-MET, FGFR1/2/3		Inhibits Aurora b kinase inhibits phosphorylation of target receptors	Advanced solid tumors			Monotherapy	ISRCTN00759419- I			Completed	Peripheral edema and hypalbuminaemia
				Recurrent glioblastoma multiforme			+ Bevacizumab	ISRCTN11619481- I/II			Completed	
ONO-7475	AXL, MER		–	Acute leukemia or MDS			Monotherapy/ + venetoclax	NCT03176277- I/II			Terminated	–
PF-07265807	AXL, MERTK		Enhance dendritic cell activity to prime CD8 + T cells	Metastatic solid tumors			Monotherapy/ + sasanlimab/ + sasanlimab and axitinib	NCT04458259- I			Active, not recruiting	–
Ningetinib	c-MET, AXL and VEGFR2		–	Stage IIIB or IV NSCLC patients with EGFR mutation and T790M-negative			+ Gefitinib	NCT03758287- I/II			Unknown status	Myocardial enzyme elevation, transaminase elevation, skin rash, diarrhea, hypertension, coagulation abnormalities, and albuminuria
CB469	AXL, c-MET		–	NSCLC			+ Erlotinib, gefitinib, osimertinib	Preclinical			–	–
Foretinib/XL880/GSK1363089	AXL, c-MET, MERTK, VEGFR1/2/3, TIE2, TYRO3, RON, FLT3, PDGFR-α/ β, c-KIT, ROS1		Inhibition of inter-receptor tyrosine kinase phosphorylation	Metastatic breast cancer			+ Lapatinib	NCT01138384-I/II			Completed	Fatigue, alopecia, diarrhea
				Recurrent/metastatic Triple-negative breast cancer			Monotherapy	NCT01147484-II			Completed	
				Papillary RCC			Monotherapy	NCT00726323-II			Completed	
				Metastatic gastric cancer			Monotherapy	NCT00725712-II			Completed	
Merestinib/LY2801653	MERTK, DDR1/2, AXL, MKNK1/2, FLT3, RON, ROS1, TYRO3, c-MET, PDGFR-α, TEK		–	Advanced or metastatic biliary tract cancer			+ Cisplatin and gemcitabine	NCT02711553-II			Active, not recruiting	Anemia, thrombocytopenia, leukopenia, neutropenia, nausea, constipation
				NSCLC harboring MET Exon 14 mutations and solid tumors with NTRK rearrangements			Monotherapy	NCT02920996-II			Terminated	
				Relapsed or refractory AML			+ LY2874455	NCT03125239-I			Completed	
				Advanced or metastatic cancer			Monotherapy/ + cisplatin and gemcitabine	NCT03027284-I			Completed	
				Advanced cancer			Monotherapy/ + cetuximab in HNSCC/ + cisplatin in cholangiocarcinoma/ + gemcitabine and cisplatin in cholangiocarcinoma/ + ramucirumab in gastric cancer	NCT01285037-I			Completed	
Gilteritinib/ASP2215	FLT3, AXL, ALK, RET, MERTK		–	FLT3/ITD AML in first complete remission			Gilteritinib vs placebo	NCT02927262-II			Completed	Febrile Neutropenia, anemia, thrombocytopenia, sepsis, pneumonia, diarrhea, fatigue
				Advanced solid tumors			Gilteritinib and 14C-labeled gilteritinib	NCT02456883-I			Completed	
				EGFRm+ advanced NSCLC with acquired resistance to an EGFR-TKI			+ Erlotinib	NCT02495233-I/II			Terminated	
Amuvatinib/MP-470	AXL-c-KIT, FLT3, c-MET, c-RET, PDGFRs		Suppresses Rad51, which can disrupt DNA repair and make tumor cells more sensitive to radiation	Solid tumors			+ paclitaxel and carboplatin/ + carboplatin and etoposide/ + topotecan/ + docetaxel/ + erlotinib	NCT00881166-I			Completed	Fatigue, alopecia, diarrhea, nausea, anorexia, neutropenia, anemia, thrombocytopenia, leukopenia
				Solid malignancies			Monotherapy	NCT00894894-I			Completed	
				Small-cell lung cancer			+ Platinum-etoposide chemotherapy	NCT01357395-II			Completed	
				Unresectable or metastatic solid tumor or lymphoma			Monotherapy	NCT00504205-I			Terminated	
MRX-2843	MERTK, FLT3, AXL		–	relapsed/refractory AML, ALL, or MPAL			Monotherapy	NCT04872478-I			Recruiting	–
Anti-AXL monoclonal antibodies (mAb) and antibody–drug conjugate (ADC)												
Tilvestamab/ BGB149	AXL mAb		Directly binds with AXL and prevents GAS6-dependent AXL activation	Relapsed, platinum-resistant, high-grade serous ovarian cancer			Monotherapy	NCT04893551- Ib			Terminated	–
YW327.6S2	AXL mAb		Binds to AXL and blocks GAS6 from binding to AXL	In (A549) NSCLC and (MDA-MB-231) breast cancer model			+ Erlotinib	Preclinical			–	–
CDX-0168	AXL mAb		Block GAS6-dependent AXL phosphorylation, stimulate ADCC, release pro-inflammatory cytokines, enhance T-cell activation	Human cancer lines and primary human myeloid cells			Monotherapy	Preclinical			–	–
Enapotamab Vedotin/ AXL-107-MMAE/ HuMax-AXL-ADC	AXL-ADC		Hinder microtubules and tubulin polymerization to block mitotic spindle assembly and induces cell-cycle arrest	Solid tumors			Monotherapy	NCT02988817- I/II			Completed	Febrile neutropenia, nausea, vomiting, constipation, diarrhea and a rise in γ-glutamyl transferase
Mipasetamab Uzoptirine/ ADCT-601	AXL-ADC		DNA interstrand cross-linking by pyrrolobenzodiazepine dimer	Solid tumors			Monotherapy/ + gemcitabine	NCT05389462-I			Recruiting	Palmar-plantar erythrodysesthesia syndrome, anemia, rash maculopapular, and cheilitis and constipation
Mecbotamab Vedotin /BA3011/ CAB-AXL-ADC	AXL-ADC		Binding to AXL only under tumor-specific conditions, such as low pH, allows the delivery of a cytotoxic agent directly to the tumor cells	Sarcoma			Mecbotamab vedotin alone / + nivolumab	NCT03425279-I/II			Recruiting	–
				NSCLC			CAB-AXL-ADC alone/ + PD-1 inhibitor	NCT04681131-II			Recruiting	
				Platinum-resistant high-grade serous ovarian cancer			+ Durvalumab	NCT04918186-II			Recruiting	
Anti-AXL-CAR therapy												
CCT301-38	AXL		Patient’s T cells are engineered to recognize and attack cancer cells with high-AXL expression	Recurrent or refractory stage IV RCC			Monotherapy	NCT03393936-I/II			Unknown status	–
				Relapsed or refractory AXL +ve sarcomas			Monotherapy	NCT05128786-I			Recruiting	
AXL-CAR-T	AXL		–	TNBC			Monotherapy	Preclinical			–	–
AXL-CAR.C7R	AXL		–	TNBC			Monotherapy	Preclinical			–	–
AXL-CAR-T	AXL		–	Advanced solid tumors			Monotherapy	NCT04842812-I			Recruiting	–
AXL-CAR-T	HER2, Mesothelin, Lewis-Y, PSCA, MUC1, GPC3, AXL, EGFR, or B7-H3		–	Advanced lung or other cancers			Monotherapy	NCT03198052-I			Recruiting	–
AXL-CAR-NK	AXL		–	Advanced solid tumors			Monotherapy/ IL7/CCL19 secreting CAR-NK cell therapy/ PD-1/PD-L1/CTLA4-scfv secreting CAR-NK cell therapy/ + Cannabidiol/ + Nicotinamide adenine dinucleotide	NCT05410717-I/II			Recruiting	–

*AML* acute myeloid leukemia, *ALL* acute lymphoblastic leukemia, *ALK* anaplastic lymphoma kinase, *BIM* Bcl-2 interacting mediator of cell death, *CLL* chronic lymphocytic leukemia, *RCC* renal cell carcinoma, *ccRCC* clear cell renal cell carcinoma, *CML* chronic myelogenous leukemia, *CML-CP* chronic myelogenous leukemia in chronic phase, *DDR* discoidin domain receptor, *EGFR* epidermal growth factor receptor, *ESCC* esophageal squamous cell carcinoma, *FLT3* Fms-like tyrosine kinase 3, *GC/GEJC* gastric/gastroesophageal junction cancer, *HCC* hepatocellular carcinoma, *HGF* hepatocyte growth factor, *HNSCC* head and neck squamous cell carcinoma, *MDS* myelodysplastic syndromes, *MPAL* mixed phenotype acute leukemia, *c-MET* mesenchymal–epithelial transition factor, *NSCLC* non-small cell lung cancer, *PDGFR* platelet-derived growth factor receptor, *ROS1* c-ros oncogene 1 receptor tyrosine kinase, *TIE2* tyrosine kinase with immunoglobulin-like and EGF-like domains 2, *TNBC* triple-negative breast cancer, *TN-IBC* triple-negative inflammatory breast cancer, *TRK* tropomyosin receptor kinase, *TKI* tyrosine kinase inhibitor, *VEGFR* vascular endothelial growth factor receptor, *XIAP* X-linked inhibitor of apoptosis protein

^a^The clinical trial and related information can be found on the public clinical trial registry website (https://www.clinicaltrials.gov/). Here, we have listed a partial list of some of the clinically relevant clinical trials

### Small-molecule AXL tyrosine kinase inhibitors (AXL TKIs)

In recent years, there has been a significant progress in the development of small-molecule kinase inhibitors that target AXL. These inhibitors work either by specifically targeting AXL or as multitargeted agents in preclinical and clinical studies.^139^ Typically, AXL has not been the primary focus of small-molecule inhibitors; instead, they target AXL due to its kinase domain’s similarities with other RTKs like c-MET. Consequently, inhibitors like cabozantinib^140^ and bosutinib,^141^ often exhibit lower potencies against AXL compared to their intended targets. Interestingly, the c-MET inhibitor BMS777607 has demonstrated greater potency against AXL than c-MET in a laboratory setting.^70^ In addition to this, several highly selective AXL-specific inhibitors have been developed, including BGB324, NA80xl, TP-0903, and SGI-7079. These belong to the category of ATP-competitive inhibitors containing an adenine-mimicking heterocyclic moiety. They bind to hinge region of ATP-binding site, either in active conformation of AXL (Aspartate-phenylalanine-glycine or D–F-G motif oriented toward the active site, DFG-in), known as ‘Type I inhibitors’^142^ or in an extended conformation by interacting with D–F-G motif through allosteric position, i.e., DFG-out conformation are known as ‘Type II inhibitors’.^143^ NPS-1034, BMS777607, cabozantinib, and sitravatinib are type II multitargeted inhibitors that are intrinsically less selective compared to type I inhibitors. These small molecules are currently at various stages of clinical trial phases and hold the potential to improve clinical outcomes for patients.^144,145^

### Anti-AXL monoclonal antibodies (mAb) and antibody–drug conjugates (ADC)

Many AXL inhibitors currently under investigation either exhibit cytotoxicity toward normal cells, lack specificity, or show only moderate effectiveness. To address these limitations, several monoclonal antibodies targeting AXL (such as Tilvestamab, YW327.6S2, CDX-0168, 20G7-D9, and MAb173) are being developed to enhance specificity and efficacy against cancer cells.^73,114,115,146,147^ Furthermore, antibody–drug conjugates (ADC) like Enapotamab Vedotin, DAXL-88, Mipasetamab Uzoptirine, and Mecbotamab Vedotin have been constructed. These ADCs deliver cytotoxic drugs via monoclonal antibodies, resulting in improved therapeutic efficacy (Table 1).^148–151^ Recently, AXL-specific single-domain antibodies (sdAbs) have demonstrated both diagnostic potential and anticancer efficacy in acute myeloid leukemia (AML).^152^ The fusion of sdAbs to an Fc domain also grants them new therapeutic capabilities, potentially enabling a multidrug strategy for treating cancer patients who test positive for AXL.

### Anti-AXL chimeric antigen receptor (CAR)-therapy

A novel cancer immunotherapy, anti-AXL chimeric antigen receptor (CAR)-T-cell therapy, has shown promise as a potential treatment option. For instance, an AXL-CAR and AXL synNotch receptors engineered using humanized single-chain variable fragment (scFv) have demonstrated the ability to target and kill tumor cells. When AXL-CAR is incorporated into human primary T cells, it enables targeted tumor cell destruction, while the AXL synNotch receptor can generate IL-10 in an antigen-specific manner.^153^ Similarly, AXL-CAR-T cells have shown antigen-specific cytotoxicity and cytokine release capacity against triple-negative breast cancer (TNBC).^154^ This therapy is precisely tailored to target AXL-positive osteomyeloid leukemic and TNBC cells,^153,154^ and is currently under clinical investigation for refractory stage IV renal cell carcinoma (RCC) (NCT03393936) and AXL-positive sarcoma (NCT05128786). However, due to the adverse side effects associated with CAR-T-cell treatment, its effectiveness in combating in battling solid tumors has been limited.^155^

To address this, AXL-CAR-T cells with constitutively activated IL7 receptor (C7R) have shown enhanced antitumor activity against TNBC cells both in vitro and in vivo, along with extended CAR-T-cell survival.^156^ In addition, the combination of AXL-CAR-T cells and microwave ablation (MWA) has exhibited improved antitumor activity in AXL-positive non-small-cell lung cancer (NSCLC) patient-derived xenograft (PDX) tumors by remodeling the tumor microenvironment (TME).^157^ AXL-CAR-T-cell immunotherapy for advanced AXL-positive lung cancer patients is currently being evaluated in phase I clinical trials to further assess its safety and efficacy (NCT03198052). Interestingly, GAS6-CAR-T cells have demonstrated effective targeting of TAM-positive pancreatic tumor cells and suppression of in vivo xenograft tumor growth with minimal toxicity by targeting both tumor cells and tumor-associated macrophages.^158^ In a recent study, Sakemura et al. found that inhibiting AXL with TP-0903 can modulate the function of CART19, enhancing its anticancer effect towards CD19+ cells through the suppression of Th2 cells and M2 macrophages. This finding suggests a novel impact of AXL inhibition on adoptive T-cell therapy’s immunological modulation.^159^ Furthermore, AXL-ADC and anti-AXL-CAR-T cells additionally showed potent and targeted cytotoxicity, improved primary tumor TME, and reduced lung metastasis in breast and lung cancer models, rendering it a plausible cancer prevention strategy.^160^ Consequently, extensive clinical research is ongoing to modify the CAR construct and explore various immune cell types as platforms to enhance the safety and effectiveness of CAR-based treatments. For instance, a phase I clinical trial involving 40 patients with AXL-positive advanced solid malignancies is underway to determine the safety and feasibility of targeting AXL with CAR-NK cells. In patients with advanced ovarian cancer or other AXL-expressing tumors, engineered CARs that target AXL are introduced into NK cells isolated from the patients’ peripheral blood.^161^ Some CAR-NK are further genetically modified to produce and release IL7/CCL19 and/or scFvs against PD-1/CTLA4/Lag3, while others are combined with cannabidiol or nicotinamide adenine dinucleotide to enhance their cytotoxic potential (NCT05410717).

## AXL-mediated acquired therapeutic resistance

Acquired resistance to current cancer therapies frequently results in unfavorable clinical outcomes, leading to tumor relapse and metastasis. Molecular studies on recurrent tumors have revealed AXL’s multifaceted role in conferring resistance to a range of treatments, including conventional and targeted therapies.^70,138^

In ovarian cancer, AXL has been identified as a key mediator of resistance to cisplatin.^162^ Similarly, acute myeloid leukemia (AML) cells from patients who developed chemotherapy resistance exhibit significantly elevated AXL expression.^108^ Additionally, AXL has been implicated in mediating resistance to cetuximab in NSCLC and head and neck squamous cell carcinoma (HNSCC),^163^ highlighting its role across various treatment modalities.^164–166^

The acquisition of secondary mutations that activate kinase signaling pathways is thought to be a primary driver of acquired therapeutic resistance, even in the presence of TKIs. These mutations can directly interfere with the effective interaction of TKIs and their target kinases by altering kinase conformation or ATP-binding affinity. While genetic alterations in the AXL gene, such as mutations (1.69%), fusions (0.04%), amplification (0.21%), or gene loss (0.09%), are relatively uncommon.^28,167^ AXL overexpression is frequently observed in multiple cancer types and is often associated with acquired therapeutic resistance.^168^

Although the precise molecular mechanisms underlying AXL-driven therapeutic resistance in the absence of genetic changes remain incompletely understood, they are thought to involve AXL-independent pathways. These may include the modulation of DNA damage and repair processes, phenotypic changes, activation of alternative signaling pathways, alterations in glucose metabolism, and interactions with other RTKs, as discussed in the following sections.

### Modulating DNA damage and DNA damage response processes

**Fig. 3:**
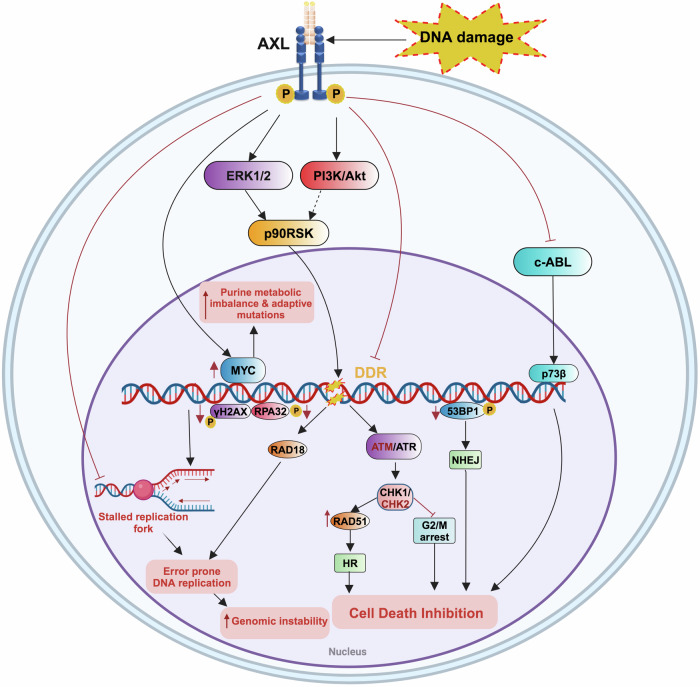
AXL induces acquired therapeutic resistance by modulating DNA damage and repair processes. AXL induces acquired therapeutic resistance by modulating DNA damage and DNA damage response processes. AXL plays a vital role in maintaining this equilibrium by inhibiting DNA damage and regulating various DNA damage response mechanisms leading to error-prone DNA replication, genomic instability and inhibition of cell death mechanisms. (HR- Homologous Recombination, NHEJ- Non-Homologous End Joining)

Numerous studies have highlighted the role of AXL signaling in orchestrating DNA damage and DNA damage response (DDR) in drug-resistant cancers. The DDR involves complex mechanisms, including repair pathways, cell-cycle checkpoints, metabolic alterations, and apoptosis induction, all of which are critical for maintaining genomic stability. When these repair pathways are dysregulated, genomic instability arises, a hallmark feature of cancer closely associated with therapy resistance (Fig. 3).^169^

Targeting AXL signaling presents a promising approach to enhance cell death by promoting DNA damage-induced activation of DDR pathways. In esophageal adenocarcinoma, AXL has been found to block apoptosis and foster drug resistance by suppressing c-ABL/p73 signaling in response to cisplatin.^170^ In addition, increased levels of AXL have been shown to stimulate ERK/mTOR signaling, triggering simultaneous DNA damage and repair signals. This promotes cell survival through CHK1 activation and confers resistance to WEE1 inhibition, a kinase involved in replication stress response.^171^

However, some studies indicate that AXL inhibition can sensitize cancer cells to other treatments. For example, study by Ramkumar et al. demonstrated that AXL inhibition activates ATR/CHK1 signaling, making NSCLC cells more susceptible to ATR inhibitors.^172^ Similarly, AXL inhibition has been observed to impede cell proliferation and induce G2/M arrest in response to DNA damage. This is evidenced by increased levels of γH2AX and other DDR markers, such as ATM/ATR/CHK2/CHK1, thereby sensitizing ovarian cancer cells to ATR inhibitors.^173^

Combining DDR and AXL signaling inhibitors has been found to have a synergistic effect in enhancing DNA damage, leading to increased cell death and improved therapy efficacy. For instance, simultaneous treatment with AXL and ATR inhibitors induces replication fork collapse and double-strand breaks (DSBs), as indicated by increased levels of phospho-RPA32 and γH2Ax proteins, ultimately leading to mitotic catastrophe.^172^ In ovarian cancer cells, combining chemotherapy with a GAS6/AXL inhibitor (AVB-500) enhances γH2AX and 53BP1 foci formation, suppresses RAD51 foci, and impedes replication fork progression compared to chemotherapy alone, thereby impairing homologous recombination repair (HRR). Conversely, the combination of a PARP inhibitor and AVB-500 accelerates replication fork progression, exacerbating DNA damage and genomic instability.^174^

Furthermore, AXL contributes to drug resistance by promoting neddylation and activation of RAD18, leading to the recruitment of low-fidelity DNA polymerases for error-prone DNA replication. This process accelerates the emergence of the T790M mutation in NSCLC cells.^30^ Additionally, AXL upregulates and activates MYC, resulting in a purine metabolic imbalance and increased adaptive mutations.^32^ In HER2+ breast cancer, replication stress response, therapy resistance, and metachronous metastasis have been linked to the nuclear AXL/WRNIP1 axis, indicating that this pathway could be therapeutically exploited.^175^ Overall, these findings highlight the complex interplay between AXL and DDR, suggesting that the simultaneous targeting of both pathways could offer significant therapeutic benefits.

### Phenotypic changes

**Fig. 4:**
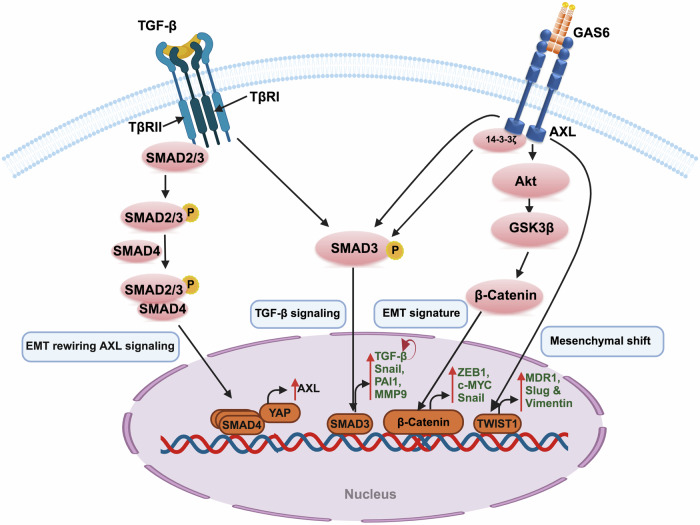
AXL-mediated phenotypic changes in cancer cells induce therapeutic resistance. AXL promotes epithelial-to-mesenchymal transition (EMT) and therapeutic resistance by inducing several EMT transcription factors, such as Snail, Slug, Smad-3, β-catenin, and TWIST1, as well as markers like MMP9, ZEB1, and Vimentin. The TGF-β/Smad-4/YAP pathway is reported to upregulate AXL expression, facilitating these phenotypic changes in cancer cells

As discussed earlier, AXL is intricately involved in phenotypic changes such as epithelial-to-mesenchymal transition (EMT), a process closely associated with the development of therapeutic resistance in cancer cells (Fig. 4).^176,177^ Increased AXL expression has been shown to upregulate vimentin while concurrently downregulating E-cadherin and the epithelial-associated miR-34a, leading to EMT and subsequent resistance to erlotinib in head and neck cancers.^178^ In addition, the GAS6/AXL axis has been reported to enhance aggressiveness and resistance to doxorubicin in breast cancer cells through the Akt/GSK-3β/β-catenin/ZEB1 pathway.^179^ Similarly, in esophageal cancer, epirubicin-induced resistance is associated with AXL-mediated transcriptional upregulation of c-MYC expression via the Akt/β-catenin signaling pathways.^180^

Furthermore, the interaction between AXL and 14-3-3ζ in hepatocellular carcinoma (HCC) leads to the phosphorylation of SMAD3 at Ser213, upregulating TGF-β target genes such as Snail, PAI1, and MMP9, and promoting autocrine TGF-β secretion.^181^ Studies on HCC further show that AXL overexpression drives metastatic progression by activating mesenchymal markers like Slug and Vimentin, contributing to sorafenib resistance.^182^ In colorectal cancer, increased AXL expression induces EMT and enhances resistance to polo-like kinase (PLK1) inhibitors via TWIST1 overexpression.^183^

Notably, the EMT state can rewire AXL signaling, further inducing drug resistance. For instance, in ovarian cancer, AXL signaling is modulated by the EMT state, with mesenchymal subtypes augmenting GAS6/AXL signaling.^184^ Similarly, TGF-β/SMAD4 activation facilitates YAP-dependent AXL expression, contributing to acquired chemoresistance in mesenchymal lung cancer cells.^185^ Further investigations into these molecular mechanisms could reveal additional targetable pathways for AXL-EMT regulation.

AXL silencing has shown potential in overcoming drug resistance. For example, using a covalent siRNA-gelatin-antibody nanoconjugate to silence AXL inhibits mTOR/EMT signaling and activates p53, thereby sensitizing NSCLC cells to TKIs.^186^ In addition, miR-625-3p-mediated targeting of AXL reverses TGF-β/SMAD driven-EMT, enhancing gefitinib sensitivity in NSCLC.^187^ Recent findings by Chen et al. demonstrated that AXL-siRNA loaded paclitaxel-poly-L-lysine prodrugs, delivered via activated T-cell-derived exosomes, can overcome AXL-mediated paclitaxel resistance and immune evasion in triple-negative breast cancer (TNBC) cells by reversing EMT and stemness processes.^188^

Given AXL’s role in inducing and maintaining the EMT phenotype, stratifying patients based on their AXL dependence is crucial. This approach could potentially reverse EMT and sensitize cancer cells to other TKIs or chemotherapeutic drugs.

### Activation of bypass signals

**Fig. 5:**
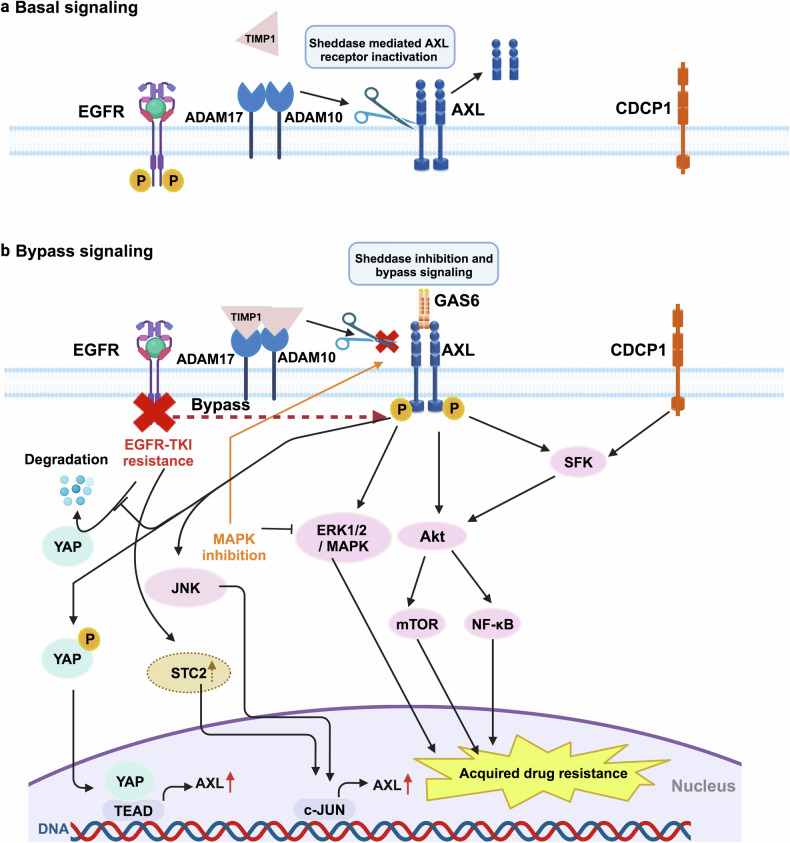
AXL-directed therapeutic resistance via activation of bypass signaling. **a** Under basal conditions, AXL is shed through the action of ADAM10/17. **b** In bypass signaling, MAPK inhibition decreases sheddase activity by enhancing the association between TIMP1 and ADAM10/17. This inhibition boosts mitogenic signaling through bypass kinases like JNK, leading to sustained secondary tolerance to EGFR TKIs

AXL has been implicated in resistance to various therapeutic agents, particularly those targeting receptor tyrosine kinases (TKIs). It maintains pathway functionality by employing alternative effectors, activating alternate signaling networks, or disrupting feedback signals (Fig. 5).^189^ In EGFR-mutant NSCLC, upregulation of AXL serves as a bypass mechanism, contributing to acquired resistance to first-generation EGFR TKIs. This resistance is partly through the activation of MAPK/ERK and PI3K/Akt pathways.^190,191^ Furthermore, Saab et al. proposed a novel perspective on how YAP and AXL contribute to EGFR-TKI resistance, amplifying signaling through a feed-forward system.^192^

Resistance to osimertinib, a third-generation EGFR-TKI, has been associated with the reactivation of multiple signaling pathways, including the AXL/CDCP1/SRC/Akt and STC2/JUN/AXL/ERK network in lung cancer.^1,193^ In addition, overexpression of AXL in NSCLC cells has been shown to induce drug-resistant persister cell phenotypes through the activation of autophagy.^194^ Another mechanism contributing to enhanced bypass signals and cancer drug resistance is extracellular proteolytic rewiring caused by blocking AXL receptor shedding.^195^ Complementing this, AXL activation and delayed degradation have been identified as critical pathways through which chemotherapeutic agents confer preserved secondary tolerance to EGFR TKIs.^196^

Given the insights, a potentially viable treatment approach for overcoming and delaying acquired resistance in cancer may involve simultaneously targeting AXL and other RTKs that share similar downstream signaling networks through combination therapy.^197,198^

### Altering glucose metabolism

**Fig. 6:**
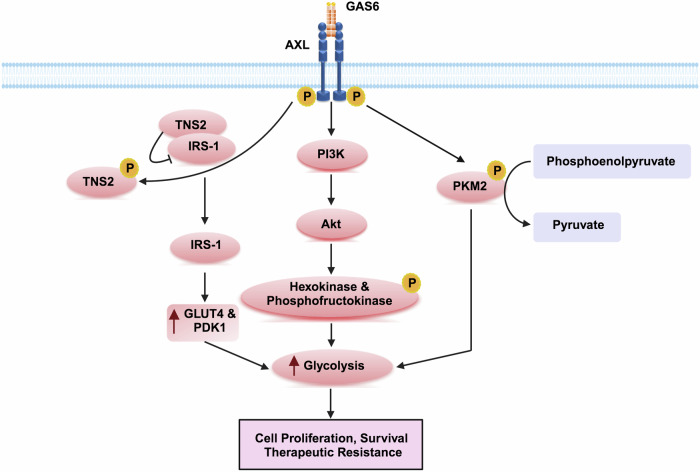
AXL alters glucose metabolism to develop acquired resistance in cancer cells. AXL-GAS6 signaling promotes glycolysis through multiple pathways, reducing sensitivity to cytotoxic stress and fostering chemoresistance. AXL-mediated PI3K/Akt signaling activates glycolysis. After AXL phosphorylates TNS2 at Y483, TNS2 becomes more stable and disengages from IRS-1. The overexpression of GLUT4 and PDK1, resulting from AXL/TNS2/IRS-1 crosstalk, enhances related metabolism. Additionally, AXL phosphorylates PKM2 at Y105, reducing its ability to bind phosphoenolpyruvate and further promoting glycolysis

Recent studies have highlighted the role of AXL in modulating glucose metabolism in cancer cells. The AXL/TNS2/IRS-1 crosstalk has been shown to influence glucose metabolism in pancreatic cancer cells by upregulating glycolytic enzymes such as Glut4 and PDK1 (Fig. 6).^199^ In the context of drug resistance, Tian et al. demonstrated that inhibiting AXL enhanced the chemosensitivity of ovarian cancer cells to cisplatin by reducing glycolysis. This effect is attributed to decreased phosphorylation of pyruvate kinase isoform M2 (PKM2) at Y105.^132^ Similarly, GAS6-AXL inhibition was found to impair the glycolytic capacity of endometrial cancer cells by reducing PI3K/Akt activation, thereby increasing sensitivity to paclitaxel.^200^

Although these studies provide a foundation, further research across different cancer types is needed to determine the precise molecular mechanisms through which AXL alters glucose metabolism to induce drug resistance.

### Crosstalk of AXL with other RTKs

AXL engages in significant crosstalk with other RTK_S_ in both the presence or absence of GAS6, amplifying pro-tumorigenic signaling. In mesenchymal ovarian cancer cells, GAS6-activated AXL forms clusters with RTKs like c-MET, HER2, and EGFR, driving motility through ERK activation and the transcription factors FRA1 and Slug.^184^ Similarly, the co-expression of AXL and PDGFR has been linked to increased invasiveness and metastasis in thyroid, esophagus, and bladder carcinomas.^201–203^

**Fig. 7:**
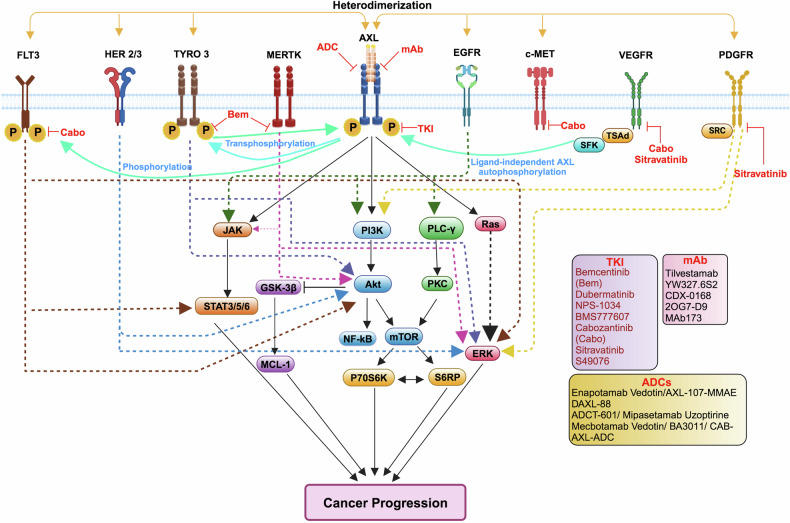
AXL crosstalk with different receptor tyrosine kinases promotes drug resistance. AXL interacts with several oncogenic receptor tyrosine kinases (RTKs), such as TYRO3 and MERTK, and heterodimerizes with several non-TAM receptors like EGFR, MET, PDGFR, and FLT3. It also forms dimers with HER2/3. These interactions activate downstream signaling pathways, leading to cancer cell proliferation, metastasis, angiogenesis, and drug resistance. Multiple tyrosine kinase inhibitors (TKIs), monoclonal antibodies (mAbs), and antibody–drug conjugates (ADCs) shown have potential in overcoming therapeutic resistance in various cancers

Recent studies have identified various mechanisms through which AXL interacts with other RTKs, to promote cancer cell survival, proliferation, invasiveness, and drug resistance. These mechanisms include heterodimerization,^204^ transphosphorylation,^204^ activation through intermediate molecules,^101^ receptor stabilization,^85^ compensation for the loss of one RTK by another,^205^ and co-expression of RTKs in specific cancers (Fig. 7).^206^ Some of them are discussed in the following sections.

#### AXL-TYRO3

TYRO3, a member of the TAM RTK family, shares the ligands GAS6 and Protein S with AXL. Although the signal transduction pathways of TYRO3 are not fully explored, its overexpression and activation have been linked to enhanced cell survival, proliferation, metastasis, and therapy resistance in various cancers.^207^

A study by Brown et al. identified a functional connection between TYRO3 and AXL, demonstrating that overexpressing kinase-active TYRO3 in Rat2 cells leads to increased GAS6-mediated AXL phosphorylation, independent of AXL expression levels. Moreover, the co-expression of TYRO3 and AXL in these cells significantly enhances proliferation, up to fivefold, upon GAS6 treatment. AXL overexpression also promotes transphosphorylation of both kinase-active and kinase-dead forms of TYRO3. This cross-phosphorylation modulates downstream of MAPK and PI3K signaling pathways, highlighting how RTK crosstalk alters cellular signaling.^202^

Both AXL and TYRO3 are overexpressed in thyroid cancer cells and leiomyosarcoma cells, contributing to cell growth and resistance to apoptosis.^208,209^ Investigating the interplay between AXL and TYRO3 in cancer progression could offer insights into the varying responses of different cancer cells to AXL inhibitors.

#### AXL-MERTK

MERTK, another member of the TAM family of RTKs, plays a physiological role in regulating innate immunity, platelet aggregation, tissue homeostasis and repair. However, its aberrant expression in various cancers has been implicated in promoting neoplasia processes such as growth factor independence, increased proliferation, resistance to apoptosis, and metastasis.^210^

While AXL and MERTK do not directly interact, their downstream signaling pathways significantly overlap. MERTK is primarily expressed in cells of the hematopoietic lineage, including monocytes, macrophages, dendritic cells, NK cells, and platelets, whereas AXL expression is mainly confined to epithelial and endothelial tissue and is linked to EMT.^99,189^ Consequently, MERTK is more closely associated with the tumor microenvironment, while AXL is linked to both tumor cells and the tumor microenvironment. This cooperation between AXL and MERTK has been shown to accelerate the growth of breast cancer cells by combining oncogenic signaling and evading host antitumor immunity.^211^

AXL and MERTK are frequently overexpressed in multiple cancers including head and neck, lung, colon, lung, skin, sarcoma, and glioblastoma multiforme.^212–216^ McDaniel et al. identified MERTK upregulation as an inherent and adaptive feedback mechanism that leads to resistance against anti-AXL agents. However, co-targeting both MERTK and AXL significantly inhibited tumor growth by affecting several signaling pathways, including Akt, STAT6, P70S6K, S6RP, and C-RAF.^215^ Interestingly, the dual inhibitor INCB081776 demonstrated immunomodulatory and antitumor effects primarily by inhibiting MERTK activation in macrophages. This effect partially counteracted M2 macrophage-mediated inhibition of T-cell proliferation and suppression of phospho-Akt.^216^

These findings suggest that co-targeting the TAM RTKs could confer dual anticancer effects. First in the tumor cells, where AXL inhibition would disrupt developmental, proliferative, and migratory pathways, and second, in the tumor microenvironment, where MERTK suppression would modulate the innate immune response.

#### AXL-c-MET

The c-MET RTK is a proto-oncogene known to derive the invasion and metastasis of various cancers.^215,217^ In clear cell renal cell carcinoma (ccRCC), GAS6/AXL signaling enhances cellular invasion through lateral stimulation of c-MET, mediated by another proto-oncogene, SRC.^57^ AXL and c-MET crosstalk has been observed in triple-negative breast cancer (TNBC), where exposure to hepatocyte growth factor (HGF) leads to phosphorylation of both c-MET and AXL. Interestingly, AXL knockdown does not affect the HGF-mediated phosphorylation of downstream proteins, indicating a complex interplay between c-MET and AXL. Conversely, GAS6 exposure results only in AXL phosphorylation, c-MET knockdown alters the timing and intensity of GAS6-mediated signaling. Despite these complexities, Reverse Phase Protein Arrays (RPPAs or RPLAs) analyses suggest that AXL and c-MET receptors exhibit crosstalk and may physically interact in breast cancer cells.^218^

In addition, Li et al. identified that the clustering of c-MET, AXL, ELMO2, and DOCK180 proteins on the plasma membrane constitutes a crucial mechanism through which HGF-dependent signaling initiates RAC1-mediated migration and invasion of glioblastoma cells.^219^ Notably, in the HGF/c-MET pathway, AXL (in its higher-molecular-weight form, p140) undergoes transphosphorylation at Tyr-779, potentially playing a significant role in tumorigenesis. This contrasts with the phosphorylation of the lower-molecular-weight AXL (p120) by GAS6 at Y702.^219^

Overall, these findings suggest that using inhibitors targeting both AXL and c-MET may be a promising strategy for preventing the progression and metastasis of cancers driven by AXL/c-MET signaling.

#### AXL-EGFR

Epidermal growth factor receptor (EGFR) is primarily involved in maintaining epithelial tissue. However, aberrant EGFR signaling can lead to epithelial transformation, making it one of the most studied tyrosine kinase receptors in cancer.^220^ Despite the clinical efficacy demonstrated by various EGFR-targeted therapies, acquired resistance often limits long-term effectiveness in cancer treatment.

Interestingly, AXL has been found to influence acquired resistance to EGFR-targeted therapies in both breast and lung cancers. In triple-negative breast cancer (TNBC), EGFR-mediated transactivation of AXL reduces responsiveness to EGFR-targeted therapies by diversifying EGFR signaling, including its interactions with c-MET and PDGFR, but not with IGF1R (insulin-like growth factor-1 receptor) or INSR (insulin receptor).^221^ In addition, co-expression of AXL and EGFR is frequently observed in lung adenocarcinomas, regardless of EGFR mutation status.^206,222^ Furthermore, crosstalk between AXL and EGFR allows squamous cell carcinomas to bypass the anti-proliferative effects of PI3Kα inhibitors by activating the PLCγ-PKC/mTOR signaling pathway.^133^ In glioblastoma multiforme, this hetero-interaction between AXL and EGFR facilitates EGFR-mediated pro-invasive signaling.^51^

Examining the co-expression patterns of EGFR and AXL in various cancers could provide valuable insights into potential biomarkers. Additionally, the concurrent targeting EGFR and AXL may enhance the therapeutic efficacy of EGFR-targeted therapies and help mitigate therapeutic resistance.

#### AXL-HER2

Another mechanism of RTK cross-communication involves the stabilization of one receptor by another, as seen with AXL and human epidermal growth factor receptor 2 (HER2). HER2, a member of the epidermal growth factor receptor family, plays a significant role in the etiology of various human cancer by regulating cellular proliferation and differentiation. Approximately 20% of breast cancer cases exhibit HER2 gene amplification and overexpression.^223^

In HER2-positive breast cancer cells, AXL physically interact with HER2, facilitating AXL’s stability and recruitment to the cell surface. This interaction leads to AXL transphosphorylation by HER2, promoting metastatic processes such as intravasation, growth of metastatic cells, and extravasation.^85^ In addition, AXL stabilizes HER2 and regulate Hypoxia-inducible factor-1 alpha (HIF-1α) levels under hypoxic conditions, potentially through activation of the PI3K/Akt pathway, which drives metastasis and modulates the tumor microenvironment in HER2-positive breast cancer cells.^224^ The crosstalk between AXL and HER2 also activates the PI3K/Akt and ERK pathways, enabling HER2-positive breast cancer cells to develop resistance against lapatinib, an inhibitor of HER2/neu and EGFR pathways.^225^

Interestingly, AXL promotes EMT and therapeutic resistance in breast cancer by heterodimerizing with HER2, thereby inducing PI3K/Akt and MAPK signaling independent of its ligands. This suggests that targeting HER2-AXL heterodimerization could be crucial for preventing HER2 inhibitor-mediated therapeutic resistance, especially in breast cancer.^137^

#### AXL-HER3

Human epidermal growth factor receptor 3 (HER3) is a member of the HER-RTK family, with neuregulins 1 (NRG1) and 2 (NRG2) as its primary ligands. Although HER3 has low intrinsic tyrosine kinase activity, it can interact with other RTK or non-RTK members to increase transphosphorylation and activate downstream pathways, ultimately promoting metastasis and drug resistance.^226^

AXL and HER3 collaborate to counteract the effects of AXL inhibitors (such as BMS777607 or R428) in MDA-MB231 and Ovcar8 cells. when AXL activity is lost, HER3 transcription is upregulated, leading to NRG1-dependent phosphorylation (Y1289) as a compensatory mechanism. Notably, the activation of phospho-Akt (S473) at low basal levels is typically required for HER3 activation upon AXL inhibition.^227^

In addition, crosstalk between AXL and HER3 promotes invasion and metastatic progression in melanoma cells by regulating invadopodia formation.^205^ In NSCLC, AXL interacts with HER3 with EGFR within a negative feedback system involving SPRY4, resulting in resistance to the EGFR inhibitor osimertinib.^222,228^ In head and neck cancer, AXL mediates cetuximab resistance through two mechanisms: activation of c-ABL kinase via AXL’s Y821 phosphorylation^229^ and HER3 activation due to upregulated NRG1.^31^

This predictive relevance of the AXL and HER3 association highlights the importance of developing dual-targeted inhibitors or combination therapies to enhance treatment efficacy against acquired resistance.

#### AXL-VEGFR

Vascular endothelial growth factor receptor (VEGFR), a member of the RTK family, plays a pivotal role in angiogenesis, with its three primary subtypes identified as VEGFR1, VEGFR2, and VEGFR3. RTKs can communicate indirectly through various signaling intermediates. One such example is how VEGFR2 activates Src family kinases via TSAd (T-cell-specific adaptor protein), promoting ligand-independent autophosphorylation of AXL at YXXM motifs. This activation facilitates interaction with PI3K, stimulating Akt signaling. Consequently, activated AXL contributes to VEGF-A/VEGFR2-dependent cancer cell migration, tube formation, vascular permeability, and corneal neovascularization.^101^

Conversely, GAS6 impedes ligand-dependent VEGFR2 activation and angiogenic signaling in vascular endothelial cells by inducing the phosphorylation of SHP-2 (Src homology region 2-containing protein tyrosine phosphatase 2) and facilitating its interaction with AXL.^230^ This intricate regulatory mechanism underscores the complexity of crosstalk between AXL and VEGFR in modulating angiogenic programs.

In addition, the localization and expression of AXL and VEGFR exhibit significant variation between monolayer and spheroid ovarian cancer models. For instance, OVCAR8 spheroids show a tenfold higher concentration of VEGFR1 on the plasma membrane and greater heterogeneity compared to monolayers, characterized by a bimodal distribution of low and high-AXL subpopulations. This variation, including a 100-fold difference in plasma membrane AXL concentration between chemo-sensitive and chemoresistance cells,^231^ highlights the importance of selecting appropriate cancer models for therapeutic screening.

In conclusion, comprehensive studies across various cancers are needed to unravel the precise molecular mechanisms underlying GAS6/AXL crosstalk with VEGFR and its regulation of angiogenic programs.

#### AXL-FLT3

The Fms-related receptor tyrosine kinase 3 (FLT3) gene, a class III RTK, plays a crucial role in hematopoiesis regulation. Upon binding to its ligand, FLT3 undergoes homodimerization on the plasma membrane, leading to autophosphorylation and initiation of downstream signaling pathways involved in cell proliferation, apoptosis, and differentiation in bone marrow. Mutations that result in constitutive activation of FLT3 are notably implicated in myeloid and lymphoblastic leukemia.^232^

AXL significantly influences the progression of FLT3 internal tandem duplication (ITD)-positive acute myeloid leukemia (AML) by promoting constitutive FLT3 phosphorylation.^233^ In FLT3-ITD positive AML cells, elevated AXL expression promotes cell growth, increases therapy resistance, and suppresses apoptosis and differentiation through regulation of downstream molecules such as Akt, STAT5, and ERK.^151,234^ Notably, inhibition of AXL activity has shown a marked reduction in the development of resistance in AML against FLT3 inhibitors, including PKC412 and AC220.^235^ Furthermore, dual inhibition of FLT3 and AXL using Gilteritinib in FLT3-ITD AML has demonstrated effectiveness in overcoming resistance mechanisms driven by the hematopoietic niche.^236^

The potential of FLT3-ITD inhibitors to induce apoptosis is influenced by the GSK-3β/Mcl-1 axis, which is regulated by both FLT3-ITD and AXL signaling pathways.^237^ Overall, combined inhibition of FLT3 and AXL presents a promising therapeutic strategy for leukemia, offering new alternatives to overcome resistance and improve patient outcomes.

#### AXL-PDGFR

Platelet-derived growth factors (PDGFs) and their receptors (PDGFRs) are expressed in various cancers, where PDGF/PDGFR signaling activation influences cell proliferation, migration, invasion, and angiogenesis through the PI3K-PKB/Akt and MAPK/ERK pathways.^238^ AXL and PDGFR demonstrate crosstalk, as observed in gastrointestinal stromal tumors (GISTs) treated with imatinib mesylate. In this context, inhibition of α-PDGFR (KIT) by imatinib mesylate leads to AXL overexpression, resulting in resistance to therapy.^239^

In human bladder cancer, c-MET has been shown to promote disease progression by transactivating the expression of AXL and PDGFR-α via MEK/ERK1/2 signaling, independent of Ras and Src.^202^ Similarly, in triple-negative breast cancer cells, AXL interacts with other ErbB receptor family members, c-MET, and PDGFR contributing to resistance against EGFR TKIs.^221^

Further exploration of the mechanisms underlying receptor switching from PDGFR to AXL in different cancers could provide valuable insights into the signaling networks and help overcome acquired resistance.

Overall, the crosstalk between AXL with other RTKs contributes to resistance against both conventional and targeted therapies across diverse cancers. Therefore, co-targeting AXL along with other RTKs presents a promising strategy to address the challenges associated with RTK signaling rewiring and improve cancer management.

## Combination therapies can potentially prevent therapeutic resistance

Given that AXL plays a crucial role in cancer progression and therapeutic resistance, combining AXL inhibitors with other receptor tyrosine kinase inhibitors offers a promising strategy for overcoming acquired resistance and enhancing drug sensitivity. Consequently, ongoing research is exploring the effectiveness of various combinations of AXL inhibitors with chemotherapies, targeted therapies, and immunotherapies. These combinations can be broadly classified into selective or multitargeted AXL inhibitor approaches.

### Selective AXL inhibitor combinations

#### Bemcentinib combinations

Bemcentinib, the first selective small-molecule AXL inhibitor to receive US FDA fast-track designation, has shown promising results in combination studies across various cancer types. Its potent inhibition of both ligand-dependent and ligand-independent AXL signaling makes it an attractive candidate for combination therapies aimed at overcoming resistance and improving treatment outcomes.

In uterine serous carcinoma, bemcentinib enhanced sensitivity to paclitaxel chemotherapy by inhibiting AXL activation.^240^ Similarly, in diffuse intrinsic pontine glioma, and aggressive colorectal adenocarcinoma, combining bemcentinib and agents like panobinostat (an HDAC inhibitor) and galunisertib (a TGF-β inhibitor) showed synergistic effects in reversing mesenchymal transition and reducing the mesenchymal phenotype, respectively.^241,242^

In NSCLC, bemcentinib combined with VX-970 (an ATR inhibitor) enhanced DNA damage and replication stress, increasing susceptibility to ATR inhibitors.^172^ In addition, in metastatic melanoma, bemcentinib improved the therapeutic efficacy of the BRAF inhibitor vemurafenib by triggering apoptosis, accelerating ferroptosis, and reducing autophagy.^243^ In older AML patients who cannot tolerate intense chemotherapy, bemcentinib showed an anti-leukemic effect when combined with low-dose cytarabine or decitabine (NCT02488408). Parallel studies have shown that combining bemcentinib with erlotinib (NCT02424617), docetaxel (NCT02922777), and pembrolizumab (a humanized antibody that targets PD-1; NCT03184571) is viable and tolerable in advanced NSCLC.

A phase Ib/II trial is currently studying the combination of bemcentinib with pembrolizumab or dabrafenib/trametinib in advanced non-resectable or metastatic myeloma (NCT02872259). Similarly, the combination of bemcentinib and pembrolizumab is being evaluated in a phase II study for mesothelioma compared to other targeted treatments (NCT03654833).

While bemcentinib/pembrolizumab has demonstrated a favorable clinical response in several trials, its investigation in refractory TNBC was halted due to the lack of complete or partial response in patients (NCT03184558). The encouraging results from these combination studies collectively support further exploration of bemcentinib in conjunction with other small-molecule inhibitors or immunotherapeutic agents for the effective treatment of various human malignancies.

#### TP-0903 combinations

In small-cell lung cancer, combining WEE1 inhibitors (such as AZD1775) and AXL inhibitors (like TP-0903, an oral selective AXL inhibitor) or with mTOR inhibitors (such as RAD001) can overcome resistance to WEE1 inhibition driven by downstream activation of mTOR and CHK1 which are involved in DNA damage repair pathways.^171^ Additionally, the combination of AXL (TP-0903) and JAK1 (ruxolitinib) inhibition has been identified as a novel therapeutic strategy based on single-cell proteomic profiling in lung cancer.^244^ Our lab has demonstrated that silencing AXL (either through gene knockdown or using TP-0903), effectively inhibits therapeutic resistance against cabozantinib in renal cell carcinoma both in vitro and in vivo.^245–247^ Moreover, targeting the heterodimerization of AXL-HER2 with TP-0903 and trastuzumab (a HER2 inhibitor) in breast cancer has been observed to overcome resistance to HER2 blockade.^137^

The preliminary clinical activity of TP-0903 in combination with decitabine has also been reported in the phase 1b/2 trial (NCT03013998) involving patients aged ≥60 years with prognostically poor TP53 mutant/complex karyotype AML.^248^ Together, these studies support the use of TP-0903 in combinations with various therapeutics to overcome the emergence of therapeutic resistance.

#### Antibody–drug conjugate combinations (CAB-AXL-ADC & Enapotamab vedotin)

Several clinical trials are investigating the safety and efficacy of antibody–drug conjugates (ADCs), such as CAB-AXL-ADC. These trials are examining its effectiveness in combination with PD-(L)1 inhibitor against various cancers, including sarcomas (phase I, NCT03425279), NSCLC (phase II, NCT04681131) and ovarian cancers (phase II, NCT04918186).

Recently, Boshuizen et al. also demonstrated the role of another AXL-targeting antibody–drug conjugate (Enapotamab vedotin) in potentiating anti-PD-1 (pembrolizumab) therapy in melanoma and lung cancer models resistant to immunotherapy. This combination results in a significant benefit from de novo immune checkpoint inhibition.^249^

### Multitargeted AXL inhibitor combinations

The development of multitargeted AXL TKI combinations has gained significant attention in recent years. These combinations aim to disrupt multiple independent significant pathways, reducing the likelihood of resistance development. For instance, TAM receptors, which include AXL, are part of complex signaling networks that being explored for combination therapies. These approaches seek to mitigate resistance to various chemotherapeutic agents and enhance treatment efficacy.^189^

#### ONO-7475 combinations

ONO-7475, a dual inhibitor targeting AXL and MERTK, has shown potential in combination therapies for overcoming resistance in various cancers. In FLT3-ITD-dependent AML, ONO-7475 can synergize with sorafenib (a multikinase inhibitor) and venetoclax (a BCL-2 inhibitor) to address resistance to FLT3 and BCL-2 inhibitors, respectively.^250,251^ In addition, combining ONO-7475 with Osimertinib (an EGFR inhibitor) has demonstrated efficacy in suppressing resistance in EGFR-mutated NSCLC cells that overexpress AXL.^252^

Despite these promising results, some clinical trials have faced challenges. The phase I/II trial (NCT03176277) exploring ONO-7475 alone or with venetoclax in AML was terminated due to protocol-defined futility criteria. Similarly, the dose-escalation study of ONO-7475 monotherapy, and in combination with anti-PD-1 therapy (ONO-4538/Nivolumab) in advanced solid tumors (NCT03730337) was also suspended following temporary completion of enrollment.

#### PF-07265807 combinations

PF-07265807, an inhibitor of AXL and MERTK, has demonstrated promising antitumor activity in preclinical models, particularly when combined with PD-1 inhibitors.^253^ A clinical trial (NCT04458259) is currently assessing the safety, tolerability, pharmacokinetics, and early antitumor efficacy of PF-07265807 as a single agent and in combination with sasanlimab (an anti-PD-1 antibody), with or without axitinib (a VEGFR inhibitor), in patients with advanced or metastatic renal cell carcinoma.^254^

In addition, Q702, a selective inhibitor targeting AXL, MERTK, and CSF1R, has shown favorable pharmacologic activity and an acceptable safety profile in patients with advanced solid tumors.^255^ This inhibitor is being evaluated in a clinical trial (NCT04648254).

Recently studies have also explored the use of AXL, TAM, and non-TAM RTK inhibitors in combination therapies to reverse acquired resistance to EGFR TKIs and improve the outcomes of immunotherapy. These combinations often show greater efficacy compared to TAM receptor-specific inhibitors alone.

#### Cabozantinib combinations

Cabozantinib, a multikinase inhibitor targeting VEGFR2, c-MET, RET, and AXL, has shown efficacy in overcoming resistance to other therapies across various cancers. In renal cancer, prolonged sunitinib treatment increases c-MET and AXL expression, leading to acquired resistance against sunitinib, which can be countered with cabozantinib.^256^ Similarly, cabozantinib combined with gefitinib has been effective in countering resistance to crizotinib and ROS1-TKIs in lung cancers with ROS1 fusions, where active HB-EGF/EGFR and AXL signaling contribute to resistance.^257^

Cabozantinib combined with osimertinib has also shown potential in overcoming AXL-mediated resistance in EGFR-mutated NSCLC.^168^ Multiple trials (NCT00596648, NCT01866410, and NCT01708954) are exploring the combination of cabozantinib with erlotinib to address EGFR-TKI resistance in NSCLC, while cabozantinib combined with panitumumab has demonstrated a favorable safety profile and encouraging activity in RAS wild-type colorectal cancer (NCT02008383).^258^

Cabozantinib is also being investigated in combination with immunotherapies. The COSMIC-021 phase 1b trial is evaluating cabozantinib alone or with atezolizumab in various solid tumors. In metastatic castration-resistant prostate cancer, cabozantinib and atezolizumab have shown a tolerable safety profile and clinically relevant activity (NCT03170960).^259^ The CONTACT-2 phase III trial reported that the combination significantly improved progression-free survival compared to abiraterone, enzalutamide, and prednisone (NCT04446117).^260^

In renal cell carcinoma, the combination of cabozantinib and nivolumab has improved overall survival compared to sunitinib and showed durable responses in advanced cases (NCT03141177).^261^ The COSMIC-313 trial showed adding cabozantinib to nivolumab and ipilimumab significantly prolonged progression-free survival in advanced renal cell carcinoma (NCT03937219).^262^ Ongoing trials, including the PDIGREE study (NCT03793166) and a phase I/II trial (NCT01658878), continue to explore these combinations, highlighting Cabozantinib’s versatility in treating various cancers and improving outcomes.

#### Sitravatinib combinations

Sitravatinib, a receptor kinase inhibitor targeting TYRO3, AXL, MERTK, and VEGFR2, is being studied in combination with nivolumab for various cancers. Ongoing clinical trials focus on its efficacy in locally advanced clear cell renal cell carcinoma (ccRCC) (NCT03015740; NCT04904302; NCT03680521), advanced NSCLC (NCT02954991; NCT03906071) and oral cancer (NCT03575598). A phase I study is also exploring the combination of sitravatinib with nivolumab and ipilimumab in ccRCC and other solid malignancies (NCT04518046). Sitravatinib combined with the anti-PD-1 antibody tislelizumab has demonstrated tolerance and early anticancer activity in patients with unresectable advanced hepatocellular carcinoma and gastric cancer (NCT03941873) and promising results in TNBC^263^ (NCT04734262), advanced melanoma^264^ and NSCLC^265^ (NCT03666143). A phase III trial is testing sitravatinib and nivolumab in advanced NSCLC (NCT04921358), while a phase II trial investigates this combination in advanced biliary tract cancer (NCT04727996). However, a phase II study in urothelial carcinoma found a tolerable safety profile but no significant objective response (NCT03606174), with 51.2% of patients experienced grade 3 treatment-related adverse events (TRAEs) 3.3% encountered grade 4 TRAEs, and one patient died from cardiac failure.^266^ The phase III SAPPHIRE trial also failed to show improved survival for sitravatinib plus nivolumab compared to docetaxel in advanced NSCLC (NCT03906071).^267^ Additional multitargeted AXL inhibitors are in development. NPS-1034, a dual inhibitor of AXL/MET, enhances gefitinib or erlotinib sensitivity in EGFR-mutant NSCLC.^268^ Ningetinib, an inhibitor of c-MET, AXL and VEGFR2 showed promise when combined with gefitinib in EGFR-TKI-resistant T790M-negative NSCLC (NCT03758287). CB469, another AXL/MET inhibitor, demonstrated efficacy with EGFR TKIs in NSCLC with acquired resistance from AXL and c-MET activation.^269^

Anlotinib combined with osimertinib has also been found to sensitize osimertinib-resistant NSCLC by targeting c-MET/MYC/AXL signaling.^270^

In addition, BMS777607, a selective MET kinase inhibitor, combined with lapatinib, overcame HER3-induced resistance to AXL TKIs.^227^ S49076, an inhibitor of MET, AXL, and FGFR, enhanced radiotherapy efficacy in MET-dependent and independent models.^271^

These studies support the ongoing exploration of AXL inhibitors in combination therapies to improve safety and overcome resistance in various cancers.

## Future prospects and conclusions

Considering AXL’s pivotal role in cancer initiation, progression, metastasis and therapeutic resistance, future research holds promise in uncovering the translational potential of AXL as a biomarker for predicting treatment responses. The numerous ongoing clinical trials involving various AXL inhibitors will help assess the clinical benefits of targeting AXL. However, several key questions about AXL remain that necessitate further investigations.

Since DNA changes are infrequent and may not reliably indicate sensitivity to AXL inhibitors, unlike targeted therapies such as EGFR inhibitors, it is crucial to explore whether the upregulated expression of AXL, often associated with poor prognosis, can serve as a reliable biomarker for identifying patients who would benefit most from AXL-targeted therapies. In addition, more research is needed to understand the epigenetic alterations governing AXL expression.

The clinical utility of AXL TKIs is limited by inherent drawbacks, such as off-target side effects resulting from the inhibition of multiple kinases. These side effects can lead to unforeseen toxicities, especially with multitargeted AXL inhibitors.^272,273^ Furthermore, the inevitability of relapse in patients with AXL-driven cancers highlights the need for high throughput screening strategies and in-depth understanding of resistance mechanisms. Such efforts are crucial to develop new treatment approaches that can mitigate potential toxicities and suppress drug resistance, resulting in more sustained therapeutic responses.

AXL’s involvement in immunological evasion and resistance to immune checkpoint blockade, mediated through regulation of the tumor microenvironment (TME), presents another significant challenge, as the survival advantage conferred by these therapies is limited to a small percentage of patients.^117^ Therefore, it is essential to delineate the mechanistic role of AXL signaling within different populations of stromal cells to better understand TME-induced antitumor defenses.

Future research should also focus on various variables affecting TME metabolism, including nutritional deprivation, pH dysregulation, hypoxia, and oxidative stress, as these factors may influence AXL expression and the selection of cells that express it.

Beyond its role in immune suppression, AXL is also implicated in EMT and cancer stemness, suggesting that inhibiting AXL could provide insights into these processes in metastatic disease and their impact on chemosensitivity and resistance. Trials investigating the potential of AXL inhibition to prevent resistance to conventional or targeted therapies are currently underway. Furthermore, non-invasive diagnostics using AXL-expressing CTC detection in future clinical studies could significantly improve personalized cancer therapy decision-making. Beyond cancer, AXL’s role in viral entry and replication (e.g., Ebola and SARS-CoV-2) and its impact on immune response modulation in comorbid conditions requires further investigation. AXL contributes to tissue homeostasis by regulating cytokine secretion and facilitating apoptotic cell clearance. Loss of AXL can lead to heightened inflammation and delayed recovery from immune-mediated damage, suggesting its potential involvement in cancer-related comorbid conditions.

Exploring the crosstalk and cooperation between AXL and other RTKs is vital for understanding the precise downstream signaling mechanisms underlying cancer cell survival. This understanding is necessary to design rational single-agent, anti-AXL-CAR or combination therapies that enhance antitumor defense and personalized treatment strategies.

In conclusion, while numerous cancer studies have demonstrated the potential of AXL as a biomarker and therapeutic target, further research is warranted to ascertain the clinical utility of AXL-based diagnostic and treatment tools.
